# Metabolic Profiling of *Cannabis* Secondary Metabolites for Evaluation of Optimal Postharvest Storage Conditions

**DOI:** 10.3389/fpls.2020.583605

**Published:** 2020-10-15

**Authors:** Looz Milay, Paula Berman, Anna Shapira, Ohad Guberman, David Meiri

**Affiliations:** The Laboratory of Cancer Biology and Cannabinoid Research, Department of Biology, Technion-Israel Institute of Technology, Haifa, Israel

**Keywords:** *Cannabis sativa* L., phytocannabinoids, terpenoids, optimal postharvest storage conditions, aging, standardization

## Abstract

The therapeutic use of medical *Cannabis* is growing, and so is the need for standardized and therapeutically stable *Cannabis* products for patients. The therapeutic effects of *Cannabis* largely depend on the content of its pharmacologically active secondary metabolites and their interactions, mainly terpenoids and phytocannabinoids. Once harvested and during storage, these natural compounds may decarboxylate, oxidize, isomerize, react photochemically, evaporate and more. Despite its widespread and increasing use, however, data on the stability of most of the plant’s terpenoids and phytocannabinoids during storage is scarce. In this study, we therefore aimed to determine postharvest optimal storage conditions for preserving the composition of naturally biosynthesized secondary metabolites in *Cannabis* inflorescences and *Cannabis* extracts. To this end, *Cannabis* inflorescences (whole versus ground samples) and *Cannabis* extracts (dissolved in different solvents) from (-)-Δ^9^-*trans*-tetrahydrocannabinol- or cannabidiol-rich chemovars, were stored in the dark at various temperatures (25, 4, −30 and −80°C), and their phytocannabinoid and terpenoid profiles were analyzed over the course of 1 year. We found that in both *Cannabis* inflorescences and extracts, a storage temperature of 25°C led to the largest changes in the concentrations of the natural phytocannabinoids over time, making this the most unfavorable temperature compared with all others examined here. Olive oil was found to be the best vehicle for preserving the natural phytocannabinoid composition of the extracts. Terpenoid concentrations were found to decrease rapidly under all storage conditions, but temperatures lower than −20°C and grinding of the inflorescences were the least favorable conditions. Overall, our conclusions point that storage of whole inflorescences and extracts dissolved in olive oil, at 4°C, were the optimal postharvest conditions for *Cannabis*.

## Introduction

*Cannabis sativa* L. (*Cannabis*) is a medicinal plant whose use dates back to several 1000 years B.C. (Mechoulam, 2019). Today, there are medical indications for *Cannabis* treatment in the fields of oncology, gastroenterology, pain management, infectious diseases, neurology, palliative care, psychiatry and more (Aggarwal et al., 2009), leading to a constant increase in the number of patients using medical *Cannabis* worldwide (Abuhasira et al., 2018).

Over 500 components from different natural product classes have been identified in the *Cannabis* plant (ElSohly and Slade, 2005). Among these, the therapeutic potential of *Cannabis* has been attributed mainly to the phytocannabinoids and terpenoids, either individually (Guzmán, 2003; Chakravarti et al., 2014; Russo and Marcu, 2017) and/or synergistically, in a phenomenon termed the “entourage effect” (Russo, 2011). Terpenoids and phytocannabinoids are both biosynthesized as secondary metabolites in the glandular trichomes, located mainly on the surface of the female inflorescence. Most phytocannabinoids are unique to *Cannabis* plants, whereas terpenoids are widespread in the plant kingdom. According to the most recent literature, over 150 phytocannabinoids and 200 terpenoids have been identified in *Cannabis* plants (Russo, 2011; ElSohly and Gul, 2014; Marchini et al., 2014; Rice and Koziel, 2015; Hanuš et al., 2016; Berman et al., 2018; Shapira et al., 2019; Nguyen et al., 2020).

Phytocannabinoids are classified into 10 subclasses according to their chemical structures, and an eleventh miscellaneous group. Among these, the cannabigerol (CBG), (-)-Δ^9^-*trans*-tetrahydrocannabinol (Δ^9^-THC), cannabidiol (CBD), and cannabichromene (CBC) subclasses are biosynthesized in *Cannabis* plants and are considered to be the main or major natural phytocannabinoids. The remaining subclasses are the result of decomposition either in the plant or as a result of light, temperature, or oxygen exposure during storage, as recently summarized by Berman et al. (2018). Phytocannabinoids are biosynthesized in the *Cannabis* plant as acids that contain a carboxyl group (COOH). Δ^9^-THC and CBD, and their respective acids, (-)-Δ^9^-*trans*-tetrahydrocannabinolic acid (Δ^9^-THCA) and cannabidiolic acid (CBDA), are the predominant phytocannabinoids and the most studied pharmacotherapeutic agents in *Cannabis* chemovars. As a result, *Cannabis* chemovars are often divided into several categories based on their phytocannabinoid contents: Type I chemovars are Δ^9^-THC-predominant, Type II contain both Δ^9^-THC and CBD, and Type III are CBD-predominant (Lewis et al., 2018).

Terpenes and terpenoids are characterized by a strong odor. Compounds from these two natural groups are volatile hydrocarbon biomolecules with diverse chemical structures. They are classified according to the number of five-carbon building blocks they contain; for example, mono- and sesqui-terpenes correspond to molecules with 10 and 15 carbons, respectively (Shapira et al., 2019). Terpenoids are modified terpenes that consist of varying oxygen arrangements or oxidation states. The general term terpenoids includes both terpenes and terpenoids (Russo, 2011).

The natural phytocannabinoids and terpenoids present in *Cannabis* are biosynthesized in the plant by specific enzymes. The compositions and concentrations of these molecules depend on the plant’s tissue-type, age, variety, growth conditions and harvest time (Berman et al., 2018; Hawley et al., 2018; Welling et al., 2018; Bernstein et al., 2019a, b; Namdar et al., 2019). Importantly, they also change over time postharvest, as a result of different degradation routes (Trofin et al., 2011, 2012; Peschel, 2016; Zamengo et al., 2019). One major example of degradation is the heat-induced decarboxylation of Δ^9^-THCA, into the psychoactive component Δ^9^-THC. All phytocannabinoid acids are susceptible to degradation when *Cannabis* is smoked, vaporized, or cooked (Dussy et al., 2005) but, importantly, the same processes can occur during storage. Terpenoids are volatile molecules and their concentration can decrease rapidly postharvest. Natural terpenoids may also decompose via isomerization, oxidation, dehydrogenation, polymerization, and thermal rearrangement (Turek and Florian, 2013). For example, some of the alcohols, ketones, and aldehydes identified in *Cannabis* have been attributed to oxidation products (Calvi et al., 2018).

**FIGURE 1:**
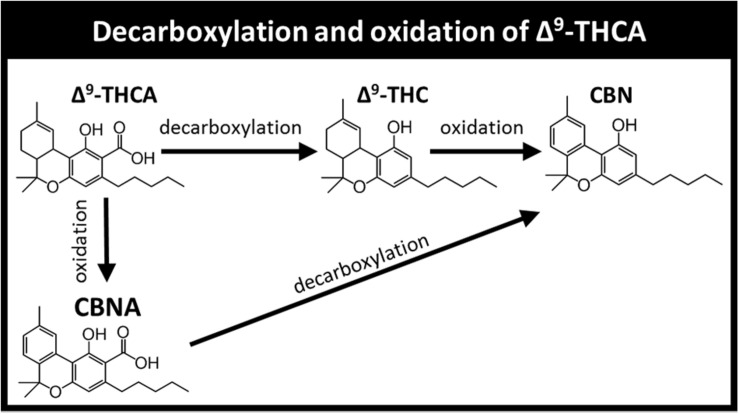
Decarboxylation and oxidation products of Δ^9^-THCA. The pathways presented include additional reactions and products not shown for brevity.

Current literature provides only limited information on the stability of phytocannabinoid and terpenoid components, and most studies focused on a few major phytocannabinoids, usually ignoring the terpenoid content. Cannabinol (CBN) for example, has been used as a marker for *Cannabis* aging in many publications (Peschel, 2016; Zamengo et al., 2019). CBN can be formed by several pathways, mainly the oxidation of Δ^9^-THC or decarboxylation of cannabinolic acid (CBNA), which in turn originates from Δ^9^-THCA oxidation (Figure 1).

One study, which focused mainly on the decarboxylation of Δ^9^-THCA to Δ^9^-THC and tested different *Cannabis* tinctures and the stability of major phytocannabinoids under two storage conditions (Peschel, 2016), found that storage in the dark at 4°C resulted in less decarboxylation of Δ^9^-THCA, CBDA, and cannabigerolic acid (CBGA) compared with storage in the light at 15–25°C. They also found that neutral phytocannabinoids stored at 4°C underwent less degradation compared with storage at higher temperatures. The authors suggested that decarboxylation is the main change occurring during long-term storage of *Cannabis* tinctures.

Another study analyzed major phytocannabinoids in *Cannabis* resin and extracts under four different storage conditions of various temperatures and light exposure (Lindholst, 2010). They found that at −20°C, the level of acidic and neutral phytocannabinoids remained constant throughout the 4-year study period, and that the major classes of acidic phytocannabinoids are more susceptible to degradation (mainly decarboxylation) than are the neutral phytocannabinoids. In this study, Δ^9^-THCA concentrations were not measured directly but rather calculated by subtracting the concentration of neutral Δ^9^-THC, as determined by high performance liquid chromatography (HPLC), from the total Δ^9^-THC concentration, as determined by gas chromatography (GC).

Two additional studies were performed on decarboxylated *Cannabis* resin and extracts (Trofin et al., 2012) and *Cannabis* inflorescences (Trofin et al., 2011). *Cannabis* resin, extracts and inflorescences were stored for 4 years at 22°C in the light and at 4°C in the dark; only Δ^9^-THC, CBD and CBN were analyzed. Nevertheless, it was found that for all *Cannabis* materials, degradation of these three neutral phytocannabinoids was higher in samples exposed to light at 22°C compared with those stored in the dark at 4°C. These findings correspond with a more recent study that used GC to analyze Δ^9^-THC, CBD, and CBN over a 4-year storage period at 22°C with and without light exposure compared with storage at 4 and −20°C in the dark (Zamengo et al., 2019).

There is some information about terpenoid stability under different storage conditions and in different plant materials (Rouseff and Naim, 2000), but almost none was published in the context of *Cannabis* storage. One such study suggests that the rate of degradation doubles for every 10°C increase in temperature, or conversely, decreases by half for each 10°C decrement (Turek and Florian, 2013).

Several of the technologies and methodologies applied in these studies are less than optimal for studying degradation and stability of *Cannabis* products. GC analyses, for example, apply high temperatures, which lead to decarboxylation of the acid phytocannabinoids in the injection port. Decarboxylation of samples prior to analysis excludes the possibility of separating and identifying acid phytocannabinoids. Furthermore, the analysis of only a limited number of compounds leads to simplistic degradation models for the different compounds.

In this study, we investigated the effect of grinding, solvent selection and storage time on phytocannabinoids and terpenoids in medical *Cannabis* inflorescences and extracts over time, in order to suggest optimal postharvest storage conditions for *Cannabis* products intended for patients or research. To this end, we applied novel methods recently developed by our group to comprehensively profile phytocannabinoids and terpenoids in such products (Berman et al., 2018; Shapira et al., 2019). Optimal postharvest storage conditions were defined as those that best preserve the composition of the naturally biosynthesized secondary metabolites in *Cannabis* inflorescences and extracts relative to their pre-storage composition.

## Materials and Methods

### Chemicals and Reagents

Liquid chromatography mass spectrometric (LC/MS) grade acetonitrile, methanol, and water for the mobile phase; ethanol, for standard solutions and sample preparation; and headspace GC grade dimethyl sulfoxide (DMSO) were purchased from Mercury Scientific and Industrial Products Ltd. (Rosh Haayin, Israel). LC/MS grade acetic acid was obtained from BioLab Ltd. (Jerusalem, Israel). Olive oil for dilution of extracts was purchased from a local distributor.

Phytocannabinoid analytical standards (> 98%) CBG, Δ^9^-THC, CBD, CBC, CBN, CBGA, Δ^9^-THCA, CBDA, CBNA, cannabichromenic acid (CBCA), (-)-Δ^8^-*trans*-tetrahydrocannabinol (Δ^8^-THC), (-)-Δ^9^-tetrahydrocannabivarin (Δ^9^-THCV), cannabidivarin (CBDV), cannabidivarinic acid (CBDVA), and cannabicyclol (CBL) were purchased from Sigma-Aldrich (Rehovot, Israel); cannabichromevarin (CBCV) was purchased from Cayman Chemical (Ann Arbor, MI, United States). Terpenoid analytical standards (> 95% unless stated otherwise) sabinene hydrate, α-terpinene, *cis*-linalool oxide, *trans*-linalool oxide, linalool, fenchol, fenchone, menthol, borneol, terpinen-4-ol, α-terpineol, citronellyl acetate, *trans*-terpin, aromadendrene, alloaromadendrene, caryophyllene oxide, and *trans*-farnesol were purchased from Sigma-Aldrich (Rehovot, Israel); valencene (> 80% pure), α- and β-curcumene (> 90% pure), α- and β-pinene, camphene, β-myrcene, α-bisabolol, β-caryophyllene, α-humulene, limonene, *trans*-nerolidol, *cis* and *trans* ocimene, γ-terpinene, and terpinolene were purchased from Restek (PA, United States); and α-phellandrene, citronellal, α- and β-curcumene, eucalyptol and sabinene were purchased from Extrasynthese (Genay, France).

### Experimental Design

The effects of storage conditions on two medical *Cannabis* chemovars with different phytocannabinoid profiles that are commonly prescribed to patients were analyzed; Type I was Δ^9^-THC-rich (Lemon Kush) and Type III was CBD-rich (Golan), according to the classification by Lewis et al. (2018). Samples from the two chemovars were stored in the dark at four different temperatures (−80, −30, 4 and 25°C) and in two physical states (whole or ground, Supplementary Figure S1A). For the ground samples, 50 g of each chemovar were ground using an electrical coffee grinder by applying ten 2 s pulses and then filtered through a 1 mm sieve. For every time point and storage temperature whole (15 g) and ground (7.5 g) samples were stored in triplicates in 100 and 20 mL bottles, respectively. Δ^9^-THC, CBN, and CBD from the two chemovars were analyzed by reversed phase ultra-high performance liquid chromatography with an ultraviolet detector (UHPLC/UV) every 4 months (t_0_, t_4_, t_8_, and t_12_ correspond to the initial time and to 4, 8, and 12 months of storage, respectively) and all phytocannabinoids were analyzed by electrospray ionization (ESI)-LC/MS at t_0_ and t_12_. Terpenoid profiles of the Type III chemovar were analyzed by static headspace (SHS)-GC/MS/MS at t_0_ and t_4_. *Cannabis* extracts were prepared by solvent extraction (Supplementary Figure S1B). Ground inflorescences from the two chemovars were extracted with ethanol, evaporated, and dissolved in either DMSO (50 mg/mL), ethanol (100 mg/mL) or olive oil (50 mg/mL). Aliquots from the same extracts were stored in the dark in amber screw top HPLC vials at the four different temperatures (−80, −30, 4 and 25°C). Phytocannabinoid analyses were performed by UHPLC/UV and ESI-LC/MS, similar to the inflorescences, every 6 months at t_0_, t_6_, and t_12_ (t_6_ corresponds with 6 months of storage). Terpenoid analyses were performed at similar time points only for the extracts dissolved in ethanol since matrix compound overlaps were observed in the developed SHS-GC/MS/MS method for both DMSO and olive oil samples.

### *Cannabis* Preparation

Inflorescences from two predominant Δ^9^-THC and CBD *Cannabis* chemovars (Types I and III, respectively) were harvested and air-dried at the farm’s drying facility for 7 days. About 150 g of the dried inflorescences were transported to the lab in sealed bags by licensed Israeli medical *Cannabis* distributors. Given that heterogeneous and variable moisture content can affect the relative content of phytocannabinoids and terpenoids in the inflorescence samples, the samples were cured prior to analysis and storage as follows: inflorescences were cured for 1 month in the dark at 21°C and 60% humidity, in 5 L sealed glass vessels that were opened every 48 h for ventilation. Moisture content of the inflorescences was analyzed immediately following the curing process and every 4 months over the course of 1 year, using an mb-50 moisture analyzer (Radwag Balances and Scales, Radom, Poland). The method used a drying temperature of 105°C, a standard heating profile, and an auto switch-off criterion (weight change of 1 mg within a 25-sec interval). Following the curing process, both chemovars contained 9 ± 0.3 % w/w moisture. In addition, periodical moisture analyses of *Cannabis* inflorescences were performed on all samples over the course of 1 year. The moisture content did not change significantly with any of the following variables: chemovar type, time of storage, storage conditions, and physical state (Supplementary Table S1). Overall, the average moisture content was 8.6 ± 0.8 % w/w.

*Cannabis* extracts were prepared by solvent extraction. Approximately 20 g of ground inflorescences from the two chemovars were extracted with 200 mL ethanol. Samples were agitated in an orbital shaker at 25°C for 15 min, and then filtered using a 500 mL Nalgene^TM^ Rapid-Flow^TM^ Sterile Disposable Filter Unit with a 0.2 μm PES membrane (Thermo Scientific, Bremen, Germany). The filtered solvent was then evaporated under reduced pressure (10 kPa for 2 h and then 5 kPa for 30 min) at 38°C using a rotary evaporator (Laborata 4000; Heidolph Instruments & Co. KG; Germany).

### Chemical Analysis of Phytocannabinoid and Terpenoid

For phytocannabinoid analysis of *Cannabis* inflorescences, 100 mg of ground *Cannabis* inflorescences were accurately weighed in triplicates and extracted with 1 mL ethanol. Samples were agitated in an orbital shaker at 25°C for 15 min, and then centrifuged at 4,200 rpm. For phytocannabinoid analysis of *Cannabis* extracts, ethanol and DMSO samples were diluted to 1 mg/mL. For the olive oil samples, 60 μL were accurately weighed and dissolved in 10 mL of the extraction solution (acetonitrile:water:acetic acid at 75:25:1 v/v). Samples were agitated in an orbital shaker at 25°C for 15 min, and then centrifuged at 4,200 rpm. All samples were filtered through a 0.22 μm PTFE syringe filter prior to analysis.

Δ^9^-THC, CBN, and CBD were analyzed by UHPLC/UV (Thermo Scientific, Bremen, Germany). Chromatographic separation was achieved using a HALO C18 Fused-Core column (2.7 μm, 150 × 2.1 mm), with a HALO guard column (2.7 μm, 5 x 2.1 mm), and a ternary A/B/C multistep gradient (solvent A: 0.1% acetic acid in water, solvent B: 0.1% acetic acid in acetonitrile, and solvent C: methanol). The multistep gradient program was established as follows: initial conditions were 50% B, which was gradually raised to 67% B over 3 min, held at 67% B for 5 min, and then raised to 90% B over the next 4 min, held at 90% B for 3 min, decreased to 50% B over the next 1 min, and finally held at 50% B for 4 min in order to re-equilibrate the system prior to the next injection. Solvent C was initially 5%, and was then lowered to 3% over the next 3 min, held at 3% for 5 min, raised to 5% over the next 4 min, and then kept constant at 5% throughout the run. Flow rate was 0.25 mL/min, column temperature was 30°C, and injection volume was 1 μL. Data acquisition was performed in full UV-Vis scan mode.

All other phytocannabinoids were identified and quantified using a similar UHPLC system coupled to a Q Exactive^TM^ Focus Hybrid Quadrupole-Orbitrap MS (Thermo Scientific, Bremen, Germany) and a similar chromatographic method as described above. Identification and absolute quantification of phytocannabinoids was performed by external calibrations, as described by Berman et al. (2018). For ESI-LC/MS analysis, the extracted samples were further diluted at ratios of 1:9, 1:99, and 1:999 v/v *Cannabis* extract to ethanol.

For terpenoid analysis, 35 mg of ground *Cannabis* inflorescences or 100 μL of ethanolic extracts were accurately weighed in triplicates. The ground samples were mixed with 100 μL LC/MS grade ethanol in 20 mL amber rounded bottom headspace vials sealed with a magnetic 32 mm PTFE septa cap. Terpenoids were analyzed using a Trace 1310 GC (Thermo Scientific, Germany) coupled to a TSQ 8000 Evo triple-quadrupole MS (Thermo Scientific, Germany), equipped with a DB-35MS UI capillary column (30 m × 0.25 mm x 0.25 μm, Agilent, United States). A CTC autosampler (Pal RTC, CTC Analytics, Switzerland) was used in SHS injection mode, with a headspace static tool in splitless mode. Identification and absolute quantification of terpenoids was performed by external calibrations, as described by Shapira et al. (2019).

### Statistical Analysis

GraphPad Prism software version 8.2.1 (GraphPad Inc.) was used to conduct the statistical analyses. Moisture contents of *Cannabis* under the different storage conditions were compared as multiple groups using two-way ANOVA, followed by a Sidak post-hoc multiple comparisons test.

Significant changes in the phytocannabinoid and terpenoid concentrations over time, temperature, physical state (whole and ground samples in the case of inflorescences), and storage solvent (DMSO, ethanol or olive oil in the case of extracts) were analyzed using the same data set (*n* = 3): time points, physical state and storage solvent were compared as multiple groups using two-way ANOVA followed by a Sidak post-hoc multiple comparisons test. Storage temperatures were compared at each time point as multiple groups using ordinary one-way ANOVA, followed by a Tukey post-hoc multiple comparisons test. A value of *p* ≤ 0.05 was considered significant for all tests.

## Results

### The Effect of Storage Parameters on the Phytocannabinoid and Terpenoid Contents of *Cannabis* Inflorescences

#### Phytocannabinoids

**FIGURE 2:**
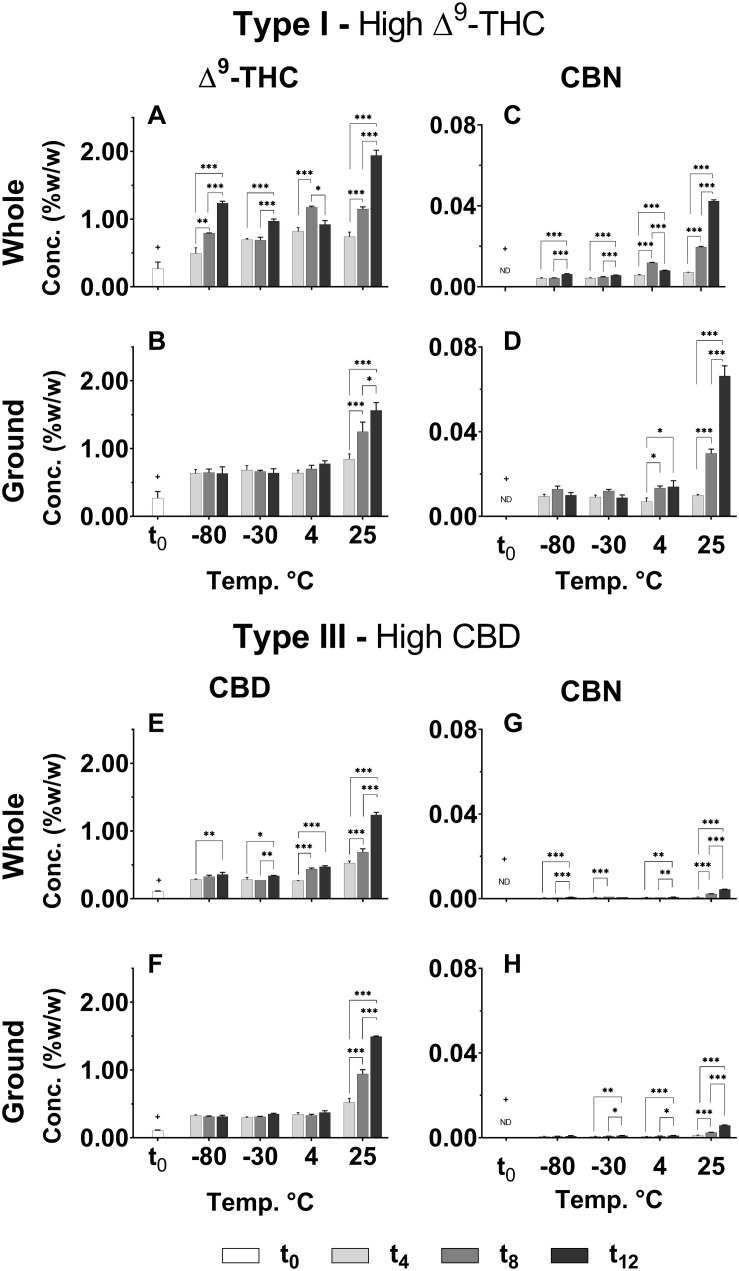
Comparison of the concentration of several major neutral phytocannabinoids in whole and ground Type I and III *Cannabis* inflorescences for various storage times and temperatures. **(A,B)** Δ^9^-THC and **(C,D)** CBN in whole and ground samples of the Type I chemovar; and **(E,F)** CBD and **(G,H)** CBN in whole and ground samples of Type III chemovar, were quantified by UHPLC/UV at 4-month intervals over the course of a year (t_0_, t_4_, t_8_, and t_12_ correspond to the initial time and 4, 8, and 12 months of storage, respectively). Data are reported as mean ± SD of phytocannabinoid concentration (*n* = 3, %w/w). Statistically significant differences between times of storage were calculated by two-way ANOVA followed by a Sidak *post hoc* multiple comparison test (*p < 0.05, **p < 0.005, ***p < 0.0005). Statistically significant differences were observed for all treatments, temperatures, and time points compared with t_0_ (+*p* < 0.0005). Statistical significant comparisons of the same data for different storage temperatures (T_1_/T_2_ of every two temperatures compared) appear in Supplementary Table S2.

As shown in Figure 2, sharp significant increases in Δ^9^-THC, CBD and CBN were observed at t_4_ (+*p* < 0.0005 for all treatments, temperatures, and time points compared with t_0_), and these continued to increase over time for all storage temperatures only for the whole inflorescences. The most significant increases in Δ^9^-THC and CBD concentrations were observed for storage at 25°C at t_12_. CBN was not detected in either of the chemovars at t_0_ and presented a similar general trend of increasing concentrations over time for all storage temperatures (Figures 2C,D,G,H for Type I and III chemovars, respectively). Importantly, this most commonly used aging marker was detected in very low concentrations in the Type III chemovar even following 1 year of storage (< 0.01% w/w in Figures 2G,H for whole and ground samples, respectively), suggesting that it is irrelevant as an aging marker for Type III chemovars.

**FIGURE 3:**
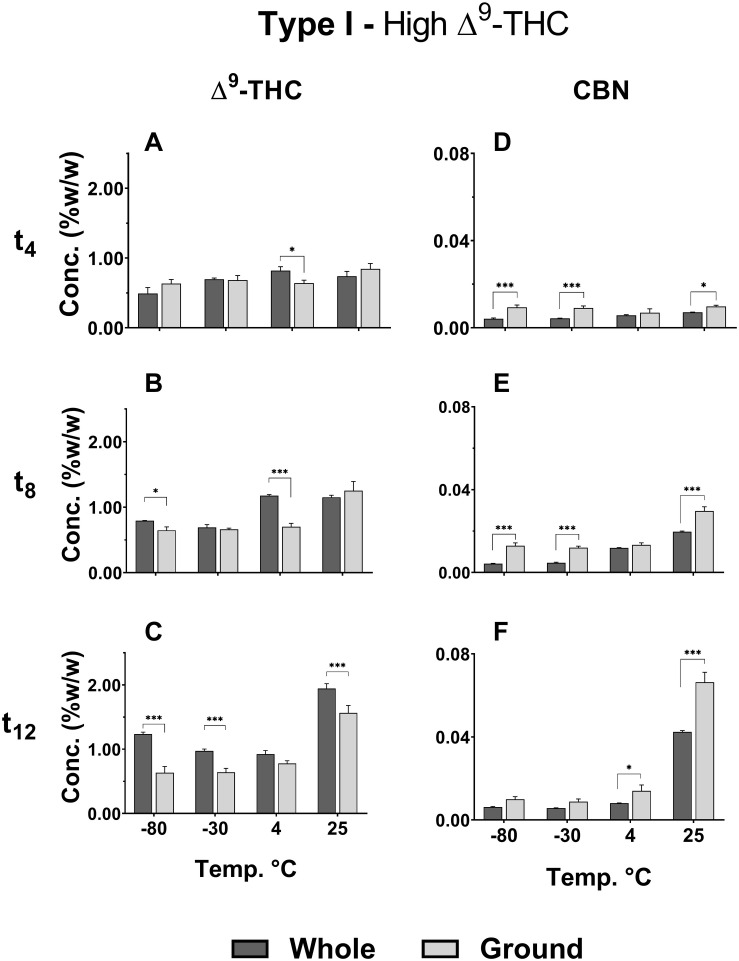
Comparison of **(A–C)** Δ^9^-THC and **(D–F)** CBN concentrations in whole and ground samples of Type I chemovar at different storage temperatures for t_4_, t_8_, and t_12_. Data are reported as mean ± SD of phytocannabinoid concentration (*n* = 3, %w/w). Statistically significant differences between whole and ground samples were calculated by two-way ANOVA followed by a Sidak post-hoc multiple comparison test (*p < 0.05, **p < 0.005, ***p < 0.0005).

Δ^9^-THC and CBD in the Type I and III ground chemovars (Figures 2B,F, respectively) did not show significant changes over time for the three colder storage temperatures (−80, −30, and 4°C), as opposed to the whole samples (Figures 2A,E for the same compounds, respectively). This may be explained by a higher rate of Δ^9^-THC decomposition compared with the rate of Δ^9^-THCA decarboxylation in the ground versus whole samples, as observed by an increase in CBN content (comparison of Δ^9^-THC and CBN of whole versus ground Type I samples appear in Figures 3A–C,D–F, respectively; Figure 1 presents the scheme for Δ^9^-THCA decarboxylation and oxidation). This higher decomposition rate may be due to a larger surface area exposure to air in the ground versus whole samples, leading to increased oxidation of phytocannabinoids.

**FIGURE 4:**
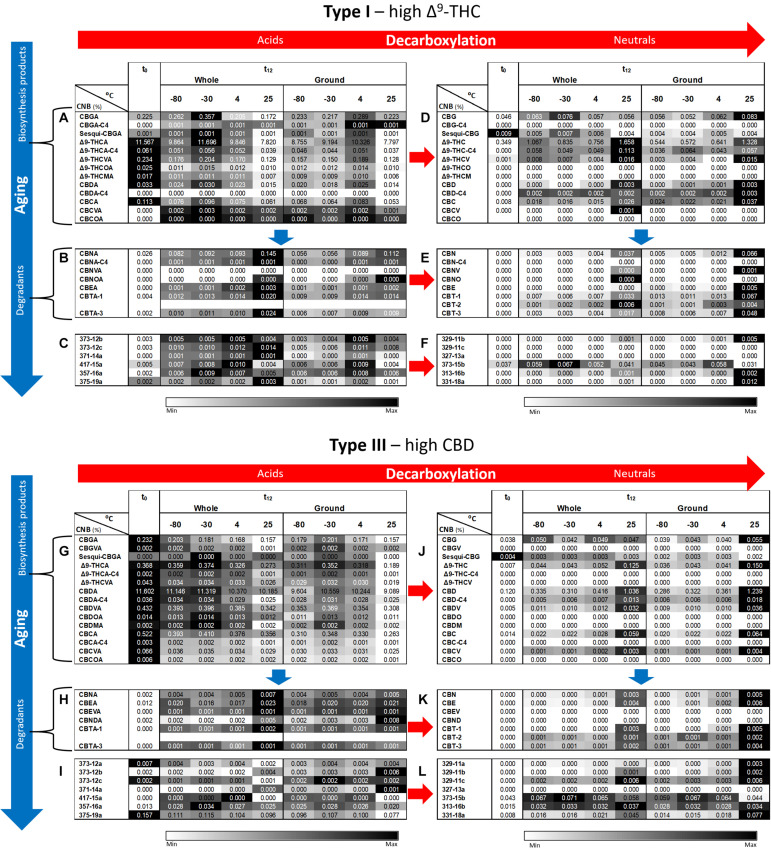
Comparison of full phytocannabinoid (CNB) profiles of whole and ground Type I and III *Cannabis* inflorescences after 1 year of storage at different temperatures. Phytocannabinoids were identified and quantified by ESI-LC/MS at the initial time and after 12 months of storage (t_0_ and t_12_, respectively). Phytocannabinoids are arranged according to biosynthesis (**A,G** for Type I and III chemovars, respectively) and degradation products (**B**–**F,H**–**L** for Type I and III chemovars, respectively). Red and blue arrows indicate decarboxylation and aging pathways, respectively. Data are reported as mean phytocannabinoid concentrations (*n* = 3, %w/w). Absolute concentrations were color coded relative to the maximum value for each compound. Supplementary Tables S4–S6, S7–S9 summarize statistical significant differences for the effects of time, temperature, and grinding for Types I and III, respectively.

Additional phytocannabinoid concentrations, including compounds with no available analytical standards, were analyzed by ESI-LC/MS (Figure 4). The full and abbreviated names of the compounds identified can be found in Supplementary Table S3. Additional phytocannabinoids whose absolute identification still remains to be resolved were identified using the names prescribed by Berman et al. (2018). As expected from the known biosynthesis and degradation pathways of phytocannabinoids (Berman et al., 2018), concentrations of most compounds biosynthesized in the plant are highest at t_0_ (Figures 4A,G for Type I and III chemovars, respectively), whereas those of most acid and neutral degradation products are lowest (Figures 4B–F,H–L for Type I and III chemovars, respectively). Aging, as indicated by significant loss of biosynthesized phytocannabinoids, was highest for samples stored at 25°C for both chemovar types and both physical states (Supplementary Tables S4A, S7A for Types I and III, respectively). For example, the Δ^9^-THCA concentrations for the Type I whole and ground samples, decreased by approximately 33 % following 1 year of storage at 25°C.

In contrast, concentrations of decarboxylated biosynthesized phytocannabinoids increased over time in Type I and III samples (Figures 4D,J, respectively), with more statistically significant changes following storage at 25°C for both chemovar types and both physical forms (Supplementary Tables S4D, S7D for Types I and III, respectively). Ambient storage temperature was also the most significantly different compared with all other storage temperatures (Supplementary Tables S5D, S8D for Types I and III, respectively). Similar to the increase in concentration of the decarboxylation products of the biosynthesized phytocannabinoids, the concentration of known oxidation and other degradation products of these phytocannabinoids and their decarboxylated compounds in Type I (Figures 4B,E, respectively) and Type III (Figures 4G,K, respectively) samples increased as well. Again, storage at 25°C resulted in more significant differences between t_0_ and t_12_ compared with the lower storage temperatures (Supplementary Tables S4B,E for Type I, and Supplementary Tables S7B,E for Type III, respectively). In most cases, the concentrations of additional unidentified phytocannabinoids (Berman et al., 2018) also increased in both Type I (Figures 4C,F) and Type III (Figures 4I,L) samples. Storage at 25°C again led to the most significant changes over time for these compounds (Supplementary Tables S4C,F for Type I, and Supplementary Tables S7C,F for Type III, respectively).

As for the effect of grinding, at t_12_ most of the biosynthesized phytocannabinoids in the ground samples exhibited a trend of reduced concentrations compared with whole ones (Figures 4A,G for the Types I and III, respectively), although this trend was not statistically significant (Supplementary Tables S6A, S9A for Type I and III chemovars, respectively). This again may be explained, as previously suggested, by the fact that grinding leads to significantly higher concentrations of many of the decomposition products compared with whole samples, especially at a storage temperature of 25°C (Supplementary Tables S6, S9 for Type I and III chemovars, respectively).

#### Terpenoids

**FIGURE 5:**
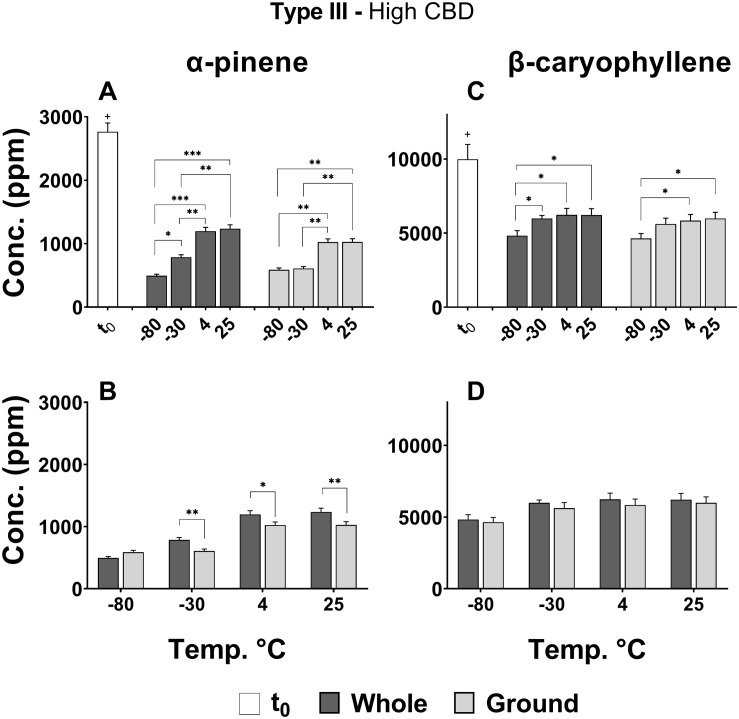
Comparison of **(A,B)** α-pinene and **(C,D)** β-caryophyllene concentrations in whole and ground Type III *Cannabis* inflorescences at t_0_ and after 4 months of storage (t_4_) at different temperatures. The two terpenoids were quantified by SHS-GC/MS/MS. Statistically significant differences between **(A,C)** storage temperatures and **(B,D)** physical form were calculated by two-way ANOVA followed by a Sidak *post hoc* multiple comparison test (*p < 0.05, **p < 0.005, ***p < 0.0005). Statistically significant differences between t_0_ and t_4_ were observed for all treatments and temperatures (+*p* < 0.0005).

Figures 5A–D show statistically significant differences in the effects of storage temperature and physical state on the concentrations of major selected mono- (α-pinene) and sesqui-terpenes (β-caryophyllene), respectively. According to the results presented, the concentrations of α-pinene (Figure 5A) and β-caryophyllene (Figure 5C) decreased significantly between t_0_ and t_4_ for all treatments and temperatures (+*p* < 0.0005). The lower storage temperatures (−80 and −30°C) had an unfavorable effect on α-pinene content compared with the other two temperatures, leading to considerable lower concentrations of this monoterpene for both whole and ground samples (Figure 5A). β-Caryophyllene (Figure 5C), on the other hand, showed mildly lower concentrations only for the −80°C storage temperature. For whole versus ground samples, only α-pinene exhibited slightly reduced concentrations following 4 months of storage (Figure 5B).

**FIGURE 6:**
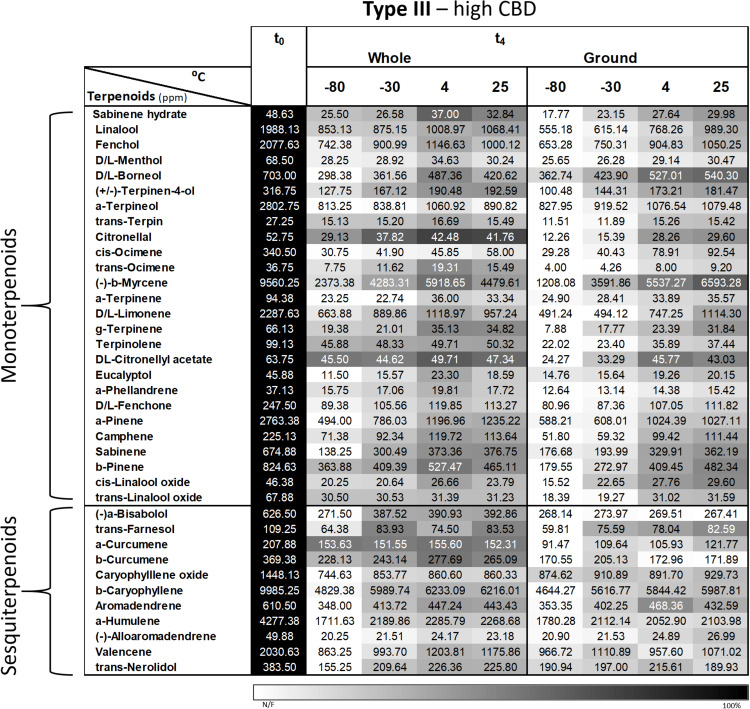
Comparison of the terpenoid profiles of whole and ground Type III *Cannabis* inflorescences after 4 months of storage (t_4_) at different temperatures. Terpenoids were identified and quantified by SHS-GC/MS/MS. Data are reported as mean terpenoid concentrations (*n* = 3, ppm). Absolute concentrations were color coded relative to the maximum value for each compound.

A more comprehensive analysis of 37 major terpenoids (of the 93 terpenoids analyzed) revealed the same general trend as α-pinene and β-caryophyllene; in other words, decreasing concentrations over time for all storage conditions (Figure 6). Importantly, a storage temperature of −80°C generally resulted in the greatest decline in terpenoid concentrations for both whole and ground samples that were stored over 4 months. In addition, lower terpenoid concentrations were observed in all of the ground samples compared with corresponding whole samples.

### The Effect of Storage Methods on the Phytocannabinoid and Terpenoid Contents of *Cannabis* Extracts

#### Phytocannabinoids

**FIGURE 7:**
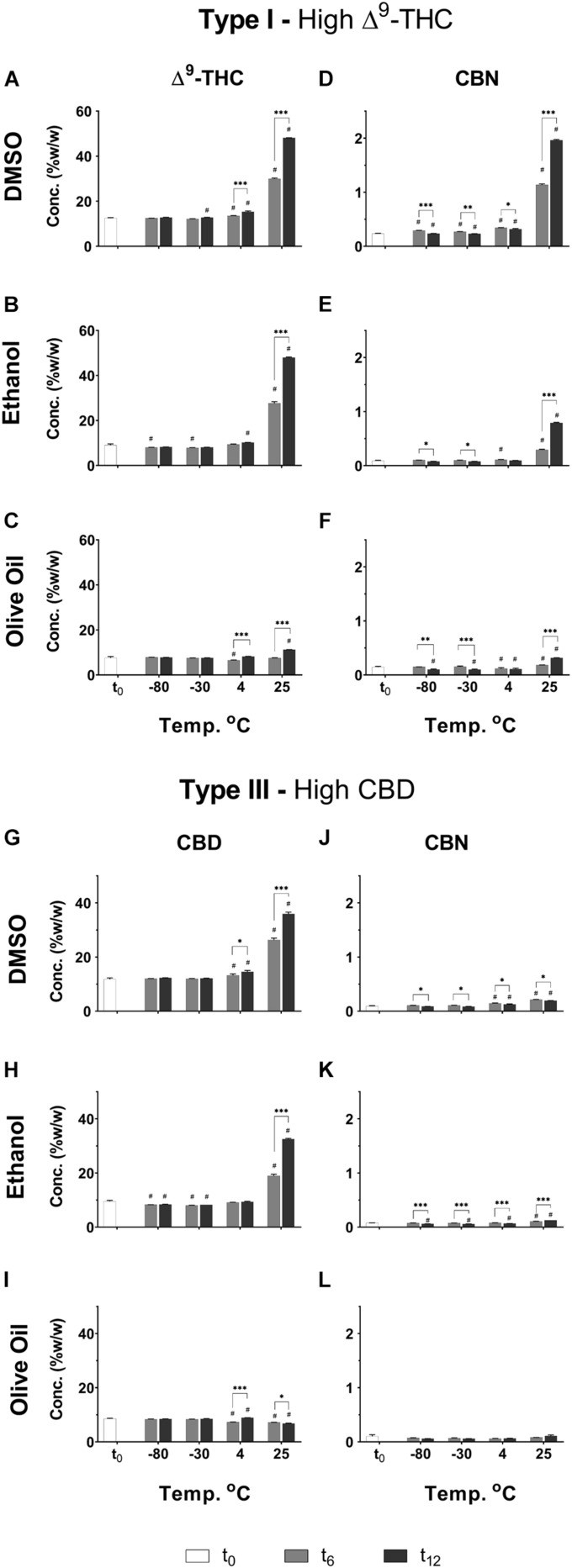
Comparison of the concentration of several major neutral phytocannabinoid in Type I and III *Cannabis* extracts dissolved and stored at different temperatures and times in either DMSO, ethanol, or olive oil. **(A–C)** Δ^9^-THC and **(D–F)** CBN in the DMSO, ethanol, and olive oil extracts of the Type I chemovar, and **(G–I)** CBD and **(J–L)** CBN in the DMSO, ethanol, and olive oil extracts of the Type III chemovar, were quantified by UHPLC/UV every 6 months over the course of a year (t_0_, t_6_, and t_12_, correspond to the initial time and then 6 and 12 months of storage, respectively). Data are reported as mean ± SD of phytocannabinoid concentration (*n* = 3, %w/w). Statistically significant differences between treatments were calculated by two-way ANOVA followed by a Sidak *post hoc* multiple comparison test (*p < 0.05, **p < 0.005, ***p < 0.0005; differences compared with t_0_: #*p* < 0.05). Supplementary Tables S10, S11 summarize statistical significant differences according to storage temperatures and solvents, respectively.

In line with the results presented for the inflorescences, significant increases were observed in Δ^9^-THC and CBD concentrations of extracts after longer storage periods at the higher storage temperatures, and especially at 25°C (Figures 7A–C,G–I, respectively). At the lower temperatures (−80 and −30°C), on the other hand, only minimal differences were observed for these compounds over time. The described effect of temperature following 1 year of storage can be further observed in Supplementary Table S10. Interestingly, at 25°C, olive oil appears to lead to less decarboxylation of Δ^9^-THC and CBD compared with the other two solvents (Supplementary Table S11).

CBN was observed at t_0_ in the Type I extracts (Figures 7D–F) probably due to some minor oxidation and/or decarboxylation of Δ^9^-THC and Δ^9^-THCA during the extraction process (Figure 1). For the Type I extract, CBN increased considerably over time especially at 25°C and, to a greater extent, in the extract dissolved in DMSO compared with the other two solvents (Figures 7D–F and Supplementary Tables S10, S11). The extracts dissolved in olive oil, on the other hand, showed very mild increases in CBN concentrations compared with t_0_ (Figure 7F), and the least significant differences at t_12_ compared with the other solvents (Supplementary Table S11).

Similar trends of increasing CBN concentrations over time and temperature are observed for Type III extracts dissolved in DMSO and ethanol (Figures 7J,K and Supplementary Tables S10, S11), although to a much lesser extent compared with Type I, as could be expected due to considerably lower concentrations of Δ^9^-THC in these extracts. No changes were observed in CBN concentrations over time for Type III extracts dissolved in olive oil (Figure 7L).

**FIGURE 8:**
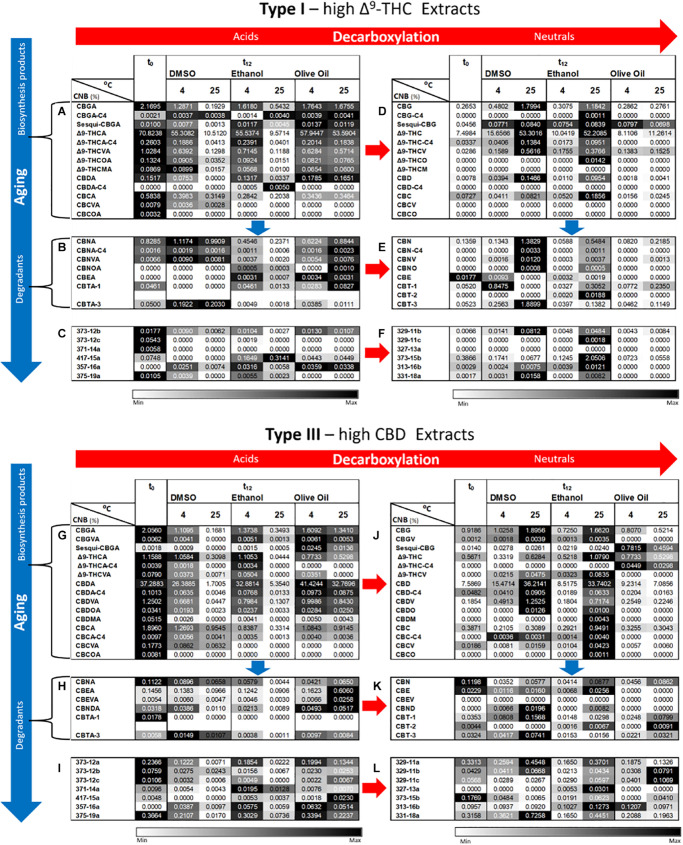
Comparison of full phytocannabinoid (CNB) profiles of Type I and III *Cannabis* extracts after 1 year of storage at 4 and 25°C in DMSO, ethanol, or olive oil. Phytocannabinoids were identified and quantified by ESI-LC/MS at the initial time and after 12 months of storage (t_0_ and t_12_, respectively). Phytocannabinoids are arranged according to biosynthesis (**A,G** for Type I and III chemovars, respectively) and degradation products (**B**–**F,H**–**L** for Type I and III chemovars, respectively). Red and blue arrows indicate decarboxylation and aging pathways, respectively. Data are reported as mean phytocannabinoid concentrations (*n* = 3, %w/w). Absolute concentrations were color coded relative to the maximum value for each compound according to storage conditions. Supplementary Tables S12–S14, S15–S17 summarize the statistical significant differences for the effect of time, temperature, and solvent for Type I and III extracts, respectively.

Figures 8A–L show additional phytocannabinoid concentrations only in extracts dissolved in the three solvents stored at 4 and 25°C. The other two cold storage temperatures analyzed showed trends close to those observed for storage at 4°C (see Supplementary Figure S2). As previously presented for the *Cannabis* inflorescences, concentrations of acid biosynthesized phytocannabinoids were generally highest at t_0_ and decreased following 1 year of storage (Figures 8A,G for Type I and III extracts, respectively). These phytocannabinoids in the *Cannabis* extracts were considerably more prone to decomposition compared with the inflorescences, for the same storage conditions (Figures 4A,G for Type I and III inflorescences, respectively). Marked decreases in the concentrations of these phytocannabinoids were observed at t_12_ with increasing storage temperature, for the extracts dissolved in DMSO and ethanol but not in olive oil (Supplementary Tables S13i–S13iii, respectively). For example, for Type I extracts stored in DMSO and ethanol, the average concentration of Δ^9^-THCA decreased at t_12_ on average by approximately 22 % and 85 % following storage at 4 and 25°C, respectively. The neutral counterpart phytocannabinoids show opposite trends (Figures 8D,J for Type I and III extracts, respectively). Following the same example, for Type I extracts stored in DMSO and ethanol, the average concentration of Δ^9^-THC increased by 170 % and 700 % following storage at 4 and 25°C, respectively. Many of the biosynthesized phytocannabinoids in the extracts stored in olive oil, on the other hand, showed slight differences in concentrations compared with t_0_, for all storage temperatures (Figures 8A,G for the acid and Figures 8D,J for the neutral biosynthesized compounds in Type I and III extracts, respectively; Supplementary Tables S12A,D, S15A,D present statistical significant differences, respectively). Specifically, following storage at 4 and 25°C, Δ^9^-THCA decreased by 18 % and 24% and Δ^9^-THC increased by 108 % and 150 %, respectively.

As expected from known degradation pathways of the major phytocannabinoids (Crombie et al., 1968; Shoyama et al., 1972; Shani and Mechoulam, 1974; Brenneisen, 2007; Carbone et al., 2010; Appendino et al., 2011; Cascini et al., 2012; Hanuš et al., 2016), decomposition products of the Δ^9^-THC subclass, including CBN and cannabitriol (CBT) derivatives, were more highly expressed in the Type I extracts, whereas those of CBD [cannabielsoin (CBE) and cannabinodiol (CBND) derivatives)] were higher in the Type III extracts (Figures 8B,E for Type I, and Figures 8H,K for Type III). Different trends were observed over time for the acid and neutral decomposition products and the different storage solvents. For Type I extracts dissolved in DMSO, for example, most major acid (Figure 8B) and neutral (Figure 8E) decomposition products showed non-significant changes in concentrations at t_12_ compared with t_0_ (Supplementary Tables S12B,E, respectively). At 25°C, on the other hand, significantly larger concentrations of the major neutral decomposition products were observed at t_12_ (Figure 8E and Supplementary Table S12E) while the acid precursors of these compounds showed mostly non-significant changes compared with t_0_ (Figure 8B and Supplementary Table S12B). For the extracts dissolved in ethanol, on the other hand, the major acid decomposition products generally exhibited lower concentrations at t_12_ compared with t_0_, and at 25°C compared with 4°C (Figure 8B and Supplementary Table S12B). The major neutral decomposition products, in contrast, had higher concentrations at 25 versus 4°C, but lower concentrations compared with the extracts in DMSO (Figure 8E and Supplementary Table S12E). For the extracts in olive oil, some increases were observed for the major acid decomposition products at 25°C at t_12_ compared with t_0_ (Figure 8B and Supplementary Table S12B). Similar trends were observed for the Type III extracts dissolved in the corresponding solvents (Figures 8H,K and Supplementary Tables S15B,E for the acid and neutral decomposition products, respectively).

#### Terpenoids

**FIGURE 9:**
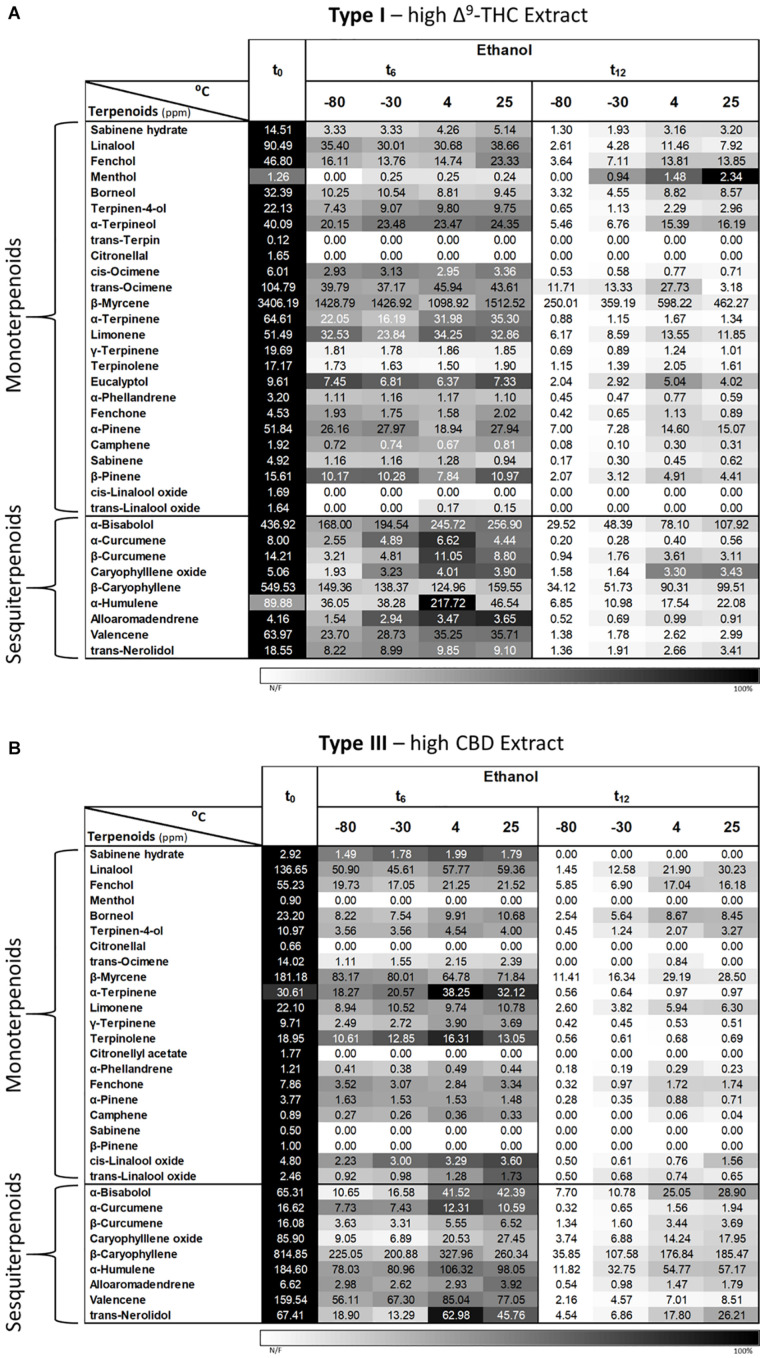
Comparison of terpenoid profiles of Type **(A)** I and **(B)** III *Cannabis* extracts dissolved and stored in ethanol for various storage times and temperatures. Terpenoids were identified and quantified by SHS-GC/MS/MS at the initial time and after 6 and 12 months of storage (t_0_, t_6_, and t_12_, respectively). Data are reported as mean terpenoid concentrations (*n* = 3, ppm). Absolute concentrations were color coded relative to the maximum value for each compound.

Since it was not possible to analyze the terpenoid contents of extracts dissolved in DMSO and olive oil due to peak overlaps between matrix/solvent and several of the analyzed compounds using the current SHS-GC/MS/MS method, we analyzed only the Type I and III extracts dissolved in ethanol (Figures 9A,B, respectively). As shown, the solvent extraction greatly reduced the absolute concentrations of most terpenoids at t_0_ compared with the corresponding inflorescences (Figures 6, 9B for Type III extract and inflorescences, respectively). This is probably due to the evaporation step in the extraction process, as previously suggested by our group (Shapira et al., 2019). Similarly to the inflorescences, a trend of reduced terpenoid concentrations over time (t_0_, t_6_ and t_12_) can be observed for both chemovar types, with slightly higher concentrations at the higher storage temperatures compared with the lower ones (4 and 25°C versus −80 and −30°C, Figure 9).

## Discussion

In this study, we found that, over time, decarboxylation was the main degradation route for all acid biosynthesized phytocannabinoids in both *Cannabis* inflorescences and extracts. This finding is in line with previous studies on *Cannabis* resin and inflorescences (Lindholst, 2010; Peschel, 2016). Although the major therapeutic knowledge on *Cannabis* focuses on phytocannabinoids in their decarboxylated form, several recent studies report on the pharmacological activities of several acid phytocannabinoids (Bolognini et al., 2013; Rock and Parker, 2013; Rock et al., 2013, 2018; Nallathambi et al., 2017; Anderson et al., 2019; Formato et al., 2020). Several *in vivo* studies even revealed that CBDA is a considerably more potent antiemetic agent than the well-studied CBD (1000 times more potent; Bolognini et al., 2013; Rock and Parker, 2013) and is also a more potent anticonvulsant agent (10 times more potent; Anderson et al., 2019). Less abundant acid phytocannabinoids (e.g., CBGA, Δ^9^-THCVA, CBDVA and others) were also found to exhibit cannabimimetic activities *in vitro* by targeting several receptors, ion channels, and/or metabolizing enzymes in the extended endocannabinoid system, as reviewed previously (Cascio and Pertwee, 2014; Di Marzo and Piscitelli, 2015).

Generally speaking, the acid phytocannabinoids in inflorescences were less prone to decarboxylation compared with the *Cannabis* extracts, for the same storage times and temperatures, although this was highly dependent on the storage solvent. Whole samples were also generally less prone to oxidation compared with ground samples. Although full decarboxylation is known to occur very rapidly at temperatures above 100°C (Dussy et al., 2005), it can also occur at ambient or lower temperatures (Wang et al., 2016; Citti et al., 2018). This is in line with the results presented here, which show that slow decarboxylation occurs over long storage periods, even at very low temperatures. A recent study analyzed the decarboxylation kinetics of CBDA dissolved in commercial hemp seed oil (Citti et al., 2018), extrapolating the half-life of CBDA to be 587 and 49 days for storage at 5°C and at 25°C, respectively. Our results show considerably longer half-lives for this compound (CBDA concentrations were 37.3 ± 3.57 % w/w at t_0_, and 41.4 ± 0.35 % and 32.8 ± 0.68 % w/w following 1 year of storage at 4 and 25°C, respectively). This result may be due to differences in the vehicle vegetable oil and warrants further investigation.

Other aging compounds analyzed include oxidation products of the major phytocannabinoids (CBN and CBT from Δ^9^-THC-type phytocannabinoids, and CBND from CBD-type phytocannabinoids), CBE-type phytocannabinoids, which are products of photochemical reactions of CBD-type compounds, and several other unknown compounds (Berman et al., 2018). The concentrations of these compounds were generally low, even for samples that exhibited the largest phytocannabinoid changes compared with t_0_. CBN, the oxidation product of Δ^9^-THC and the most widely used marker for determining *Cannabis* aging, exhibited a general trend of increasing concentrations with time and temperature for both chemovars. We also observed increases in CBN concentrations over time for ground versus whole samples and for extracts dissolved in DMSO versus ethanol and olive oil. Total CBN concentrations (the sum of CBN and CBNA concentrations) in the *Cannabis* samples naturally correlated also to total Δ^9^-THC content for the same storage conditions, as can be observed for example for the Type I versus Type III chemovars. Concentrations of both CBN and CBNA can, therefore, serve as aging markers for high-potency chemovars, but only in relation to their initial Δ^9^-THC and Δ^9^-THCA contents. Following the same rule, this marker is irrelevant for Type III chemovars.

Concentrations of terpenoids in inflorescences, which also possess therapeutic potentials of their own or in combination with phytocannabinoids (Russo and Marcu, 2017), were considerably lower compared with t_0_, for all temperatures, even after only 4 months of storage. By calculating the relative losses of all terpenoids and biosynthesized phytocannabinoids compared with t_0_, (total average of the ratio between the concentration of each compound under a specific storage condition and its corresponding concentration at t_0_, as presented in Figures 4G, 6, respectively) we found that the average terpenoid concentration decreased by more than 50% at t_4_ as opposed to an average 26% loss for all the biosynthesized phytocannabinoids at t_12_. Surprisingly, despite their very different volatilities, similar trends were observed for both mono- and sesqui-terpenoids. According to these results, it is very difficult to preserve the original (t_0_) terpenoid contents in inflorescences, and this must be accounted for when reporting the terpenoid concentrations of a specific chemovar. *Cannabis* extracts also exhibited terpenoid losses, although less dominant compared with the inflorescences, probably due to the lower terpenoid concentrations at t_0_ that are the result of evaporation during the chemical extraction process.

Phytocannabinoids in *Cannabis* inflorescences are more stable during storage compared with extracts under similar conditions. As shown here, the solvent chosen as the vehicle for dissolving the *Cannabis* extract can also have unfavorable effects on its stability. DMSO was the least favorable of the three solvents analyzed, while olive oil was the best preservative, probably due to its known conserving characteristics (Cheung et al., 2007; Serra et al., 2008). DMSO and ethanol are often used in research labs as the *Cannabis* extract vehicle for challenging cells and/or animals in different biological models. According to the presented results, *Cannabis* extracts used in long-term studies should be analyzed periodically to detect major changes in their chemical compositions. Since temperature is a major factor in phytocannabinoids degradation, extracts should be aliquoted to avoid numerous freeze-thaw cycles.

In this study we found that the best conditions for preserving the original phytocannabinoid and terpenoid contents of inflorescences throughout long storage periods are as whole inflorescences at 4°C. In regards to medical *Cannabis* oil preparation and storage, *Cannabis* inflorescences intended for extraction should be stored under these optimal conditions, and then extracted as close as possible to the oil’s marketing, dissolved in olive oil, and stored at 4°C. It should be noted, however, that olive oil freezes at this temperature, and so the oil should be consumed only after re-melting and proper mixing to avoid phase separation.

The therapeutic use of medical *Cannabis* is growing, and so is the need for standardized and therapeutically stable *Cannabis* products for patients. Prolonged storage under sub-optimal conditions can lead to degradation or changes in biologically active phytocannabinoids and terpenoids resulting in new constituents. This can consequently alter the therapeutic effects of the *Cannabis* medication or lead to undesirable side effects compared with the original material. This study, therefore, provides important information that is relevant to *Cannabis* growers, clinicians, manufacturers, and distributors of medical *Cannabis*, in order to provide patients with standardized, pharmaceutical-grade *Cannabis* inflorescences and oils.

## Data Availability Statement

All datasets generated for this study are included in the article/Supplementary Material.

## Author Contributions

LM performed all of the experiments and analyzed the data. AS performed the SHS-GC/MS/MS analyses and analyzed the data. OG performed the extraction and sample preparation of the *Cannabis* extracts. LM, PB, and DM contributed to the design of the experimental plan. LM and PB drafted the manuscript. DM led and coordinated the overall project. All authors proofread and approved the final manuscript.

## Conflict of Interest

The authors declare that the research was conducted in the absence of any commercial or financial relationships that could be construed as a potential conflict of interest.
