# Supplementary material for: Metabolic Profiling of Cannabis Secondary Metabolites for Evaluation of Optimal Postharvest Storage Conditions
Source: Front Plant Sci. 2020 Oct 15;11:583605. doi: 10.3389/fpls.2020.583605 (PMC7593247; doi:10.3389/fpls.2020.583605)
Supplement: Supplementary file 1 [file Data_Sheet_1.docx]

Supplementary Material

**Metabolic profiling of *Cannabis* secondary metabolites for evaluation of optimal postharvest storage conditions**

**Looz Milay^1†^, Paula Berman^1†^, Ohad Guberman^1^, Anna Shapira^1^, David Meiri^1*^**

^1^The Laboratory of Cancer Biology and Cannabinoid Research, Department of Biology, Technion-Israel Institute of Technology, Haifa 3200003, Israel

^†^These authors contributed equally to this publication.

*** Correspondence:**David Meiri
[dmeiri@technion.ac.il](mailto:dmeiri@technion.ac.il)


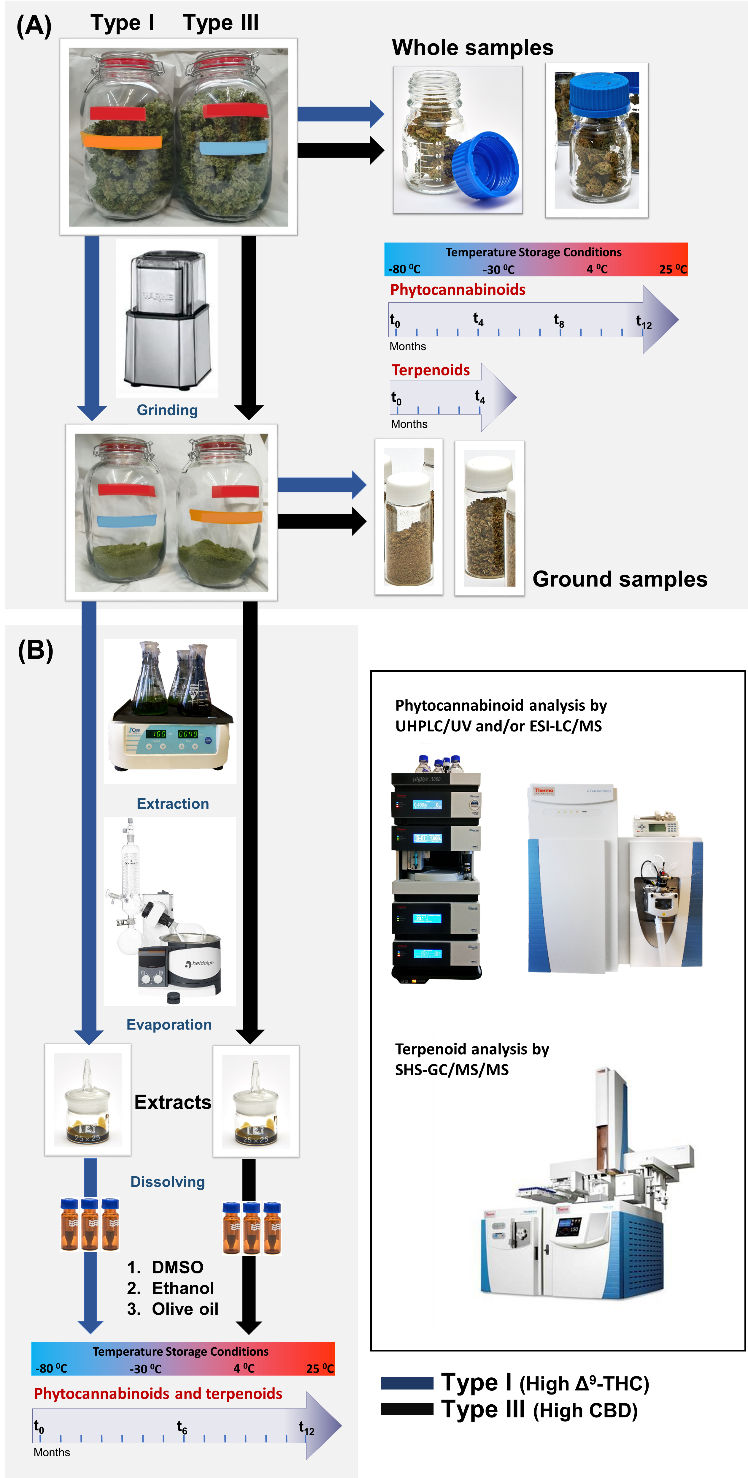


**Figure S1. Experimental design for the treatment of *Cannabis* inflorescences and extracts and the analytical procedures performed at each time point.** **(A)** Samples from two chemovars (Types I and III) were stored in the dark at four different temperatures (-80, -30, 4 and 25 ºC) in two physical states (whole or ground). Δ^9^-THC, CBN, and CBD from the two chemovars were analyzed by UHPLC/UV every 4 months (t_0_, t_4_, t_8_, and t_12_ correspond to the initial time and 4, 8, and 12 months of storage, respectively) and all phytocannabinoids were analyzed by ESI-LC/MS at t_0_ and t_12_. Terpenoid profiles of the Type III chemovar were analyzed by SHS-GC-MS/MS at t_0_ and t_4_. **(B)** Ground inflorescences from the two chemovars were extracted with ethanol, evaporated, and dissolved in either DMSO, ethanol, or olive oil at a concentration of 50 mg/mL (100 mg/mL for ethanol), and stored in the dark at four different temperatures (-80, -30, 4 and 25 ºC). Phytocannabinoids were analyzed at t_0_, t_6_, and t_12_ as described previously. Terpenoid analyses were performed at similar time points only for extracts dissolved in ethanol.


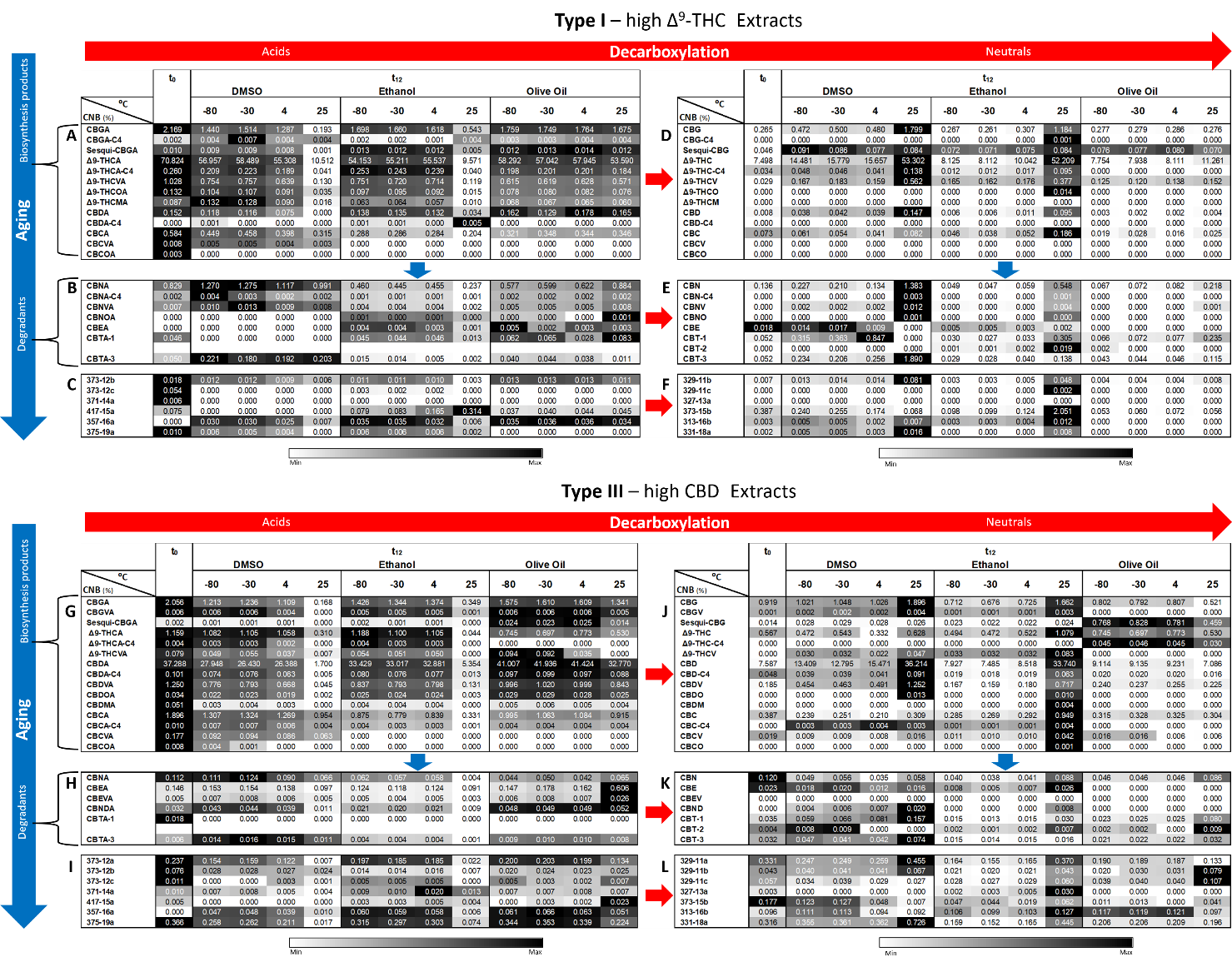


Figure S2. Comparison of full phytocannabinoid (CNB) profiles of Type I and III *Cannabis* extracts following one year of storage at different temperatures in DMSO, ethanol or olive oil. Phytocannabinoids were identified and quantified by ESI-LC/MS at the initial time and following 12 months of storage (t_0_ and t_12_, respectively). Phytocannabinoids are arranged by biosynthesis (A and G for Type I and III chemovars, respectively) and degradation products (B-F and H-L for Type I and III chemovars, respectively). Red and blue arrows indicate decarboxylation and aging pathways, respectively. Data are reported as mean phytocannabinoid concentrations (n=3, %w/w). Absolute concentrations were color coded relative to the maximum value of each compound according to storage conditions.

**Table S1. Statistical analysis of the moisture content over time of *Cannabis* inflorescences**

| **Test^a^** | **Sub test** | **P-Value** | **Summary** |
| --- | --- | --- | --- |
| Type I vs Type III | - | 0.9906 | ns |
| Type I vs Type III | Whole | >0.9999 | ns |
| Type I vs Type III | Ground | 0.7067 | ns |
| Type I vs Type III | -80⁰C | >0.9999 | ns |
| Type I vs Type III | -30⁰C | 0.9998 | ns |
| Type I vs Type III | 4⁰C | 0.4264 | ns |
| Type I vs Type III | 25⁰C | 0.9812 | ns |
| Whole vs Ground | - | 0.9837 | ns |
| t_4_ vs t_8_ | - | 0.2211 | ns |
| t_4_ vs t_12_ | - | 0.6705 | ns |
| t_8_ vs t_12_ | - | 0.8280 | ns |

^a^Moisture contents of *Cannabis* inflorescences under the different storage conditions were compared as multiple groups using two-way ANOVA, followed by a Sidak post-hoc multiple comparisons test. ns: not significant.

**Table S2. Statistical significant differences in the concentration of major neutral phytocannabinoid (CNB) in whole and ground samples at different storage times for different storage temperature (T_1_/T_2_)**

|  |  |  | **Whole** | | | | | | **Ground** | | | | | |
| --- | --- | --- | --- | --- | --- | --- | --- | --- | --- | --- | --- | --- | --- | --- |
| **Time** | **Chemovar Type** | **CNB** | **Comparison of storage temperatures^a^ [°C]** | | | | | | **Comparison of storage temperatures^a^ [°C]** | | | | | |
|  |  |  | **25/4** | **25/-30** | **25/-80** | **4/-30** | **4/-80** | **-30/-80** | **25/4** | **25/-30** | **25/-80** | **4/-30** | **4/-80** | **-30/-80** |
| **t_4_** | I | Δ^9^-THC |  |  | * |  | ** | * | * |  | * |  |  |  |
|  |  | CBN | * | *** | *** | ** | ** |  |  |  |  |  |  |  |
|  |  | CBD |  |  |  |  |  |  |  |  |  |  |  |  |
|  | III | Δ^9^-THC | *** | *** | *** |  |  |  | * | ** | * |  |  |  |
|  |  | CBN |  |  | ** |  | * | * | * | * | * |  |  |  |
|  |  | CBD | *** | *** | *** |  |  |  | * | ** | ** |  |  |  |
| **t_8_** | I | Δ^9^-THC |  | *** | *** | *** | *** | * | *** | *** | *** |  |  |  |
|  |  | CBN | *** | *** | *** | *** | *** |  | *** | *** | *** |  |  |  |
|  |  | CBD |  |  |  |  |  |  |  |  |  |  |  |  |
|  | III | Δ^9^-THC | *** | *** | *** | * | * |  | *** | *** | *** |  |  |  |
|  |  | CBN | *** | *** | *** | * |  | ** | *** | *** | *** |  |  |  |
|  |  | CBD | *** | *** | *** | ** | * |  | *** | *** | *** |  |  |  |
| **t_12_** | I | Δ^9^-THC | *** | *** | *** |  | ** | * | *** | *** | *** |  |  |  |
|  |  | CBN | *** | *** | *** | ** | ** |  | *** | *** | *** |  |  |  |
|  |  | CBD |  |  |  |  |  |  |  |  |  |  |  |  |
|  | III | Δ^9^-THC | *** | *** | *** | * | * |  | *** | *** | *** |  |  |  |
|  |  | CBN | *** | *** | *** |  |  |  | *** | *** | *** |  |  |  |
|  |  | CBD | *** | *** | *** | * | * |  | *** | *** | *** |  | * |  |

^a^One-way ANOVA followed by a Tukey post-hoc multiple comparisons test. A value of p ≤ 0.05 was considered significant for all tests.

**Table S3. List of full and abbreviated names of phytocannabinoids**

| **1. Cannabigerol (CBG) type** | **Abbreviated name** |
| --- | --- |
| Cannabigerol-C5 | CBG |
| CBG-C4 | CBG-C4 |
| Cannabigerovarin-C3 | CBGV |
| CBG-C1 | CBGO |
| Cannabigerol monomethyl ether-C5 | CBGM |
| Sesquicannabigerol-C5 | SesquiCBG |
| Cannabigerolic acid-C5 | CBGA |
| CBGA-C4 | CBGA-C4 |
| Cannabigerovarinic acid-C3 | CBGVA |
| CBGA-C1 | CBGOA |
| Cannabigerolic acid monomethyl ether-C5 | CBGMA |
| Sesquicannabigerolic acid-C5 | SesquiCBGA |
| **2. Δ^9^-*trans*-tetrahydrocannabinol (Δ^9^-THC) type** | **Abbreviated name** |
| (-)-Δ^9^-*trans*-Tetrahydrocannabinol-C5 | Δ^9^-THC |
| (-)-Δ^9^-*trans*-Tetrahydrocannabinol-C4 | Δ^9^-THC-C4 |
| (-)-Δ^9^-*trans*-Tetrahydrocannabivarin-C3 | Δ^9^-THCV |
| (-)-Δ^9^-*trans*-Tetrahydrocannabiorcol-C1 | Δ^9^-THCO |
| Δ^9^-THCM-C5 | Δ^9^-THCM |
| (-)-Δ^9^-*trans*-Tetrahydrocannabinolic acid-C5 | Δ^9^-THCA |
| (-)-Δ^9^-*trans*-Tetrahydrocannabinolic acid-C4 | Δ^9^-THCA-C4 |
| (-)-Δ^9^-*trans*-Tetrahydrocannabivarinic acid-C3 | Δ^9^-THCVA |
| (-)-Δ^9^-*trans*-Tetrahydrocannabiorcolic acid-C1 | Δ^9^-THCOA |
| Δ^9^-THCMA-C5 | Δ^9^-THCMA |
| **3. Cannabidiol (CBD) type** | **Abbreviated name** |
| (-)-Cannabidiol-C5 | CBD |
| Cannabidiol-C4 | CBD-C4 |
| (-)-Cannabidivarin-C3 | CBDV |
| Cannabidiorcol-C1 | CBDO |
| Cannabidiol monomethyl ether-C5 | CBDM |
| Cannabidiolic acid-C5 | CBDA |
| CBDA-C4 | CBDA-C4 |
| Cannabidivarinic acid-C3 | CBDVA |
| Cannabidiorcolic acid-C1 | CBDOA |
| CBDMA-C5 | CBDMA |
| **4. Cannabichromene (CBC) type** | **Abbreviated name** |
| (±)-Cannabichromene-C5 | CBC |
| CBC-C4 | CBC-C4 |
| (±)-Cannabichromevarin-C3 | CBCV |
| Cannabiorchromene-C1 | CBCO |
| (±)-Cannabichromenic acid-C5 | CBCA |
| CBCA-C4 | CBCA-C4 |
| (±)-Cannabichromevarinic acid-C3 | CBCVA |
| Cannabiorchromenic acid-C1 | CBCOA |
| **5. Cannabinol (CBN) type** | **Abbreviated name** |
| Cannabinol-C5 | CBN |
| Cannabinol-C4 | CBN-C4 |
| Cannabivarin-C3 | CBNV |
| Cannabiorcol-C1 | CBNO |
| 8-OH-CBN-C5 | OH-CBN |
| Cannabinol methyl ether-C5 | CBNM |
| Cannabinolic acid-C5 | CBNA |
| Cannabinolic acid-C4 | CBNA-C4 |
| CBNA-C3 | CBNVA |
| Cannabiorcolic acid-C1 | CBNOA |
| 8-OH-CBNA-C5 | OH-CBNA |
| **6. Δ^8^-trans-tetrahydrocannabinol (Δ^8^-THC) type** | **Abbreviated name** |
| (-)-Δ^8^-*trans*-(6aR,10aR)-Tetrahydrocannabinol-C5 | Δ^8^-THC |
| **7. Cannabicyclol (CBL) type** | **Abbreviated name** |
| (±)-(1aS,3aR,8bR,8cR)-Cannabicyclol-C5 | CBL |
| **8. Cannabinodiol (CBND) type** | **Abbreviated name** |
| Cannabinodiol-C5 | CBND |
| Cannabinodiolic acid-C5 | CBNDA |
| CBNDA-C3 | CBNDVA |
| **9. Cannabielsoin (CBE) type** | **Abbreviated name** |
| (5aS,6S,9R,9aR)-Cannabielsoin-C5 | CBE |
| Cannabielsoin-C3 | CBEV |
| (5aS,6S,9R,9aR)-Cannabielsoic acid-C5 | CBEA |
| Cannabielsoic acid-C3 | CBEVA |
| **10. Cannabitriol (CBT) type** | **Abbreviated name** |
| (±)-*cis/trans*-Cannabitriol-C5-1 | CBT-1 |
| (±)-*cis/trans*-Cannabitriol-C5-2 | CBT-2 |
| (±)-*cis/trans*-Cannabitriol-C5-3 | CBT-3 |
| Cannabitriol-C3-1 | CBTV-1 |
| Cannabitriol-C3-3 | CBTV-3 |
| (±)-*cis/trans*-Cannabitriolic acid-C5-1 | CBTA-1 |
| (±)-*cis/trans*-Cannabitriolic acid-C5-3 | CBTA-3 |
| **Additional phytocannabinoids** | **Abbreviated name** |
| Cannabicitran | Cannabicitran |

**Table S4. Statistical significant differences in phytocannabinoid concentrations in whole and ground samples of the Type I chemovar stored at different temperatures for different storage times (t_0_ versus t_12_^a^)**

|  |  | **Whole** | | | | **Ground** | | | |  |  |  | **Whole** | | | | **Ground** | | | |
| --- | --- | --- | --- | --- | --- | --- | --- | --- | --- | --- | --- | --- | --- | --- | --- | --- | --- | --- | --- | --- |
|  |  | **Storage Temperature [°C]** | | | | **Storage Temperature [°C]** | | | |  |  |  | **Storage Temperature [°C]** | | | | **Storage Temperature [°C]** | | | |
|  |  | **-80** | **-30** | **4** | **25** | **-80** | **-30** | **4** | **25** |  |  |  | **-80** | **-30** | **4** | **25** | **-80** | **-30** | **4** | **25** |
| **(A)** | **CBGA** |  | * |  |  |  |  |  |  |  | **(D)** | **CBG** | ** | *** | * | * |  |  |  | ** |
|  | **CBGA-C4** |  | * | ** | ** |  |  | * | * |  |  | **CBG-C4** |  |  |  |  |  |  |  |  |
|  | **SesquiCBGA** |  |  |  |  |  |  |  |  |  |  | **SesquiCBG** | *** | * | ** | *** | *** | *** | ** | *** |
|  | **Δ^9^-THCA** |  |  |  | ** | * |  |  | * |  |  | **Δ^9^-THC** | * | * |  | *** |  |  | * | *** |
|  | **Δ^9^-THCA-C4** | * |  | * | *** |  | * |  | * |  |  | **Δ^9^-THC-C4** |  |  |  | * |  | * |  | * |
|  | **Δ^9^-THCVA** | * |  | * | ** | * | * |  | * |  |  | **Δ^9^-THCV** |  |  |  | * |  |  |  | * |
|  | **Δ^9^-THCOA** | *** | *** | *** | *** | *** | *** | ** | *** |  |  | **Δ^9^-THCO** |  |  |  |  |  |  |  |  |
|  | **Δ^9^-THCMA** | *** | *** | *** | *** | *** | *** | ** | *** |  |  | **Δ^9^-THCM** |  |  |  |  |  |  |  |  |
|  | **CBDA** | * |  | * | *** | * | * |  | ** |  |  | **CBD** |  |  |  | * |  |  |  | ** |
|  | **CBDA-C4** |  |  |  |  |  |  |  |  |  |  | **CBD-C4** |  |  |  |  |  |  |  |  |
|  | **CBCA** | *** | * | *** | *** | ** | ** | * | *** |  |  | **CBC** | *** | ** | * | *** | * | * | * | *** |
|  | **CBCVA** | *** | *** | *** | *** | *** | *** | *** | * |  |  | **CBCV** |  |  |  |  |  |  |  |  |
|  | **CBCOA** |  |  |  |  |  |  |  |  |  |  | **CBCO** |  |  |  |  |  |  |  |  |
| **(B)** | **CBNA** | ** | *** | *** | *** | * | * | ** | *** |  | **(E)** | **CBN** |  |  | * | *** |  |  | * | *** |
|  | **CBNA-C4** |  |  |  | *** |  |  |  | *** |  |  | **CBN-C4** |  |  |  |  |  |  |  |  |
|  | **CBNVA** |  |  |  |  |  |  |  |  |  |  | **CBNV** |  |  |  | *** |  |  |  | *** |
|  | **CBNOA** |  |  |  | *** |  |  |  | * |  |  | **CBNO** |  |  |  |  |  |  |  |  |
|  | **CBEA** |  |  | * | * |  |  | * | * |  |  | **CBE** |  |  |  |  |  |  |  |  |
|  | **CBTA-1** | *** | *** | *** | *** | * | * | ** | ** |  |  | **CBT-1** | * |  | * | *** | * | * | * | *** |
|  |  |  |  |  |  |  |  |  |  |  |  | **CBT-2** | * | * | * | *** | * |  | *** | *** |
|  | **CBTA-3** | ** | ** | ** | *** | * | * | ** | ** |  |  | **CBT-3** |  |  |  | *** |  |  |  | * |
| **(C)** | **373-12b** | * | * | ** | * |  |  | * |  |  | **(F)** | **329-11b** |  |  |  | * |  |  |  | ** |
|  | **373-12c** | ** | *** | *** | *** |  |  | *** | * |  |  | **329-11c** |  |  |  |  |  |  |  |  |
|  | **371-14a** |  |  |  |  |  |  |  |  |  |  | **327-13a** |  |  |  |  |  |  |  |  |
|  | **417-15a** |  |  |  |  |  |  |  |  |  |  | **373-15b** | ** | *** | * |  |  |  | * |  |
|  | **357-16a** | * | ** | ** | * | * | * | * | * |  |  | **313-16b** |  |  |  |  |  |  |  |  |
|  | **375-19a** |  |  |  |  |  |  |  |  |  |  | **331-18a** |  |  |  |  |  |  |  |  |

^a^One-way ANOVA followed by a Tukey post-hoc multiple comparisons test. A value of p ≤ 0.05 was considered significant for all tests.

**Table S5. Statistical significant differences in phytocannabinoid concentrations in whole and ground samples of the Type I chemovar following one year of storage for different storage temperatures (T_1_/T_2_)**

|  |  | **Whole** | | | | | | **Ground** | | | | | |  |  |  | **Whole** | | | | | | **Ground** | | | | | |
| --- | --- | --- | --- | --- | --- | --- | --- | --- | --- | --- | --- | --- | --- | --- | --- | --- | --- | --- | --- | --- | --- | --- | --- | --- | --- | --- | --- | --- |
|  |  | **Comparison of storage temperatures^a^ [°C]** | | | | | | **Comparison of storage temperatures^a^ [°C]** | | | | | |  |  |  | **Comparison of storage temperatures^a^ [°C]** | | | | | | **Comparison of storage temperatures^a^ [°C]** | | | | | |
|  |  |  |  |  |  |  |  |  |  |  |  |  |  |  |  |  |  |  |  |  |  |  |  |  |  |  |  |  |
|  |  | **25/4** | **25/**  **-30** | **25/**  **-80** | **4/**  **-30** | **4/**  **-80** | **-30/**  **-80** | **25/4** | **25/**  **-30** | **25/**  **-80** | **4/**  **-30** | **4/**  **-80** | **-30/**  **-80** |  |  |  | **25/4** | **25/**  **-30** | **25/**  **-80** | **4/**  **-30** | **4/**  **-80** | **-30/**  **-80** | **25/4** | **25/**  **-30** | **25/**  **-80** | **4/**  **-30** | **4/**  **-80** | **-30/**  **-80** |
| **(A)** | **CBGA** |  | ** | * | * |  | * |  |  |  |  |  |  |  | **(D)** | **CBG** |  | *** | * | *** |  | * | * | * | * |  |  |  |
|  | **CBGA-C4** |  |  |  |  |  |  |  |  |  | * |  |  |  |  | **CBG-C4** |  |  |  |  |  |  |  |  |  |  |  |  |
|  | **SesquiCBGA** |  |  |  |  |  |  |  |  |  |  |  |  |  |  | **SesquiCBG** | * | ** | * | * |  | * |  |  |  |  |  |  |
|  | Δ^9^-**THCA** | * | ** | * | * |  | * |  |  |  |  |  |  |  |  | Δ^9^-**THC** | ** | * | * |  |  |  | *** | *** | *** |  |  |  |
|  | Δ^9^-**THCA-C4** | * | ** | * |  |  |  |  |  |  |  |  |  |  |  | Δ^9^-**THC-C4** |  |  |  |  |  |  |  |  |  |  |  |  |
|  | Δ^9^-**THCVA** |  | * | * |  |  |  |  |  |  |  |  |  |  |  | Δ^9^-**THCV** |  |  |  |  |  |  | * | * | * |  |  |  |
|  | Δ^9^-**THCOA** |  | ** |  | * |  | * |  |  |  |  |  |  |  |  | Δ^9^-**THCO** |  |  |  |  |  |  |  |  |  |  |  |  |
|  | Δ^9^-**THCMA** | * | ** | * |  |  |  | * |  | * |  |  |  |  |  | Δ^9^-**THCM** |  |  |  |  |  |  |  |  |  |  |  |  |
|  | **CBDA** | * | * | * | * |  |  | * |  |  |  |  |  |  |  | **CBD** |  | * |  |  |  |  | * | * | * |  |  |  |
|  | **CBDA-C4** |  |  |  |  |  |  |  |  |  |  |  |  |  |  | **CBD-C4** |  |  |  |  |  |  |  |  |  |  |  |  |
|  | **CBCA** |  | ** | * | * |  | * | * |  |  |  |  |  |  |  | **CBC** | *** | *** | ** |  |  |  | * | * | * |  |  |  |
|  | **CBCVA** |  | * |  |  |  |  | * |  | * |  |  |  |  |  | **CBCV** |  |  |  |  |  |  |  |  |  |  |  |  |
|  | **CBCOA** |  |  |  |  |  |  |  |  |  |  |  |  |  |  | **CBCO** |  |  |  |  |  |  |  |  |  |  |  |  |
| **(B)** | **CBNA** | ** | ** | *** |  |  |  |  | ** | ** |  | * |  |  | **(E)** | **CBN** | *** | *** | *** |  |  |  | *** | *** | *** |  |  |  |
|  | **CBNA-C4** | * | * | * |  |  |  |  | * |  |  |  |  |  |  | **CBN-C4** |  |  |  |  |  |  |  |  |  |  |  |  |
|  | **CBNVA** |  |  |  |  |  |  |  |  |  |  |  |  |  |  | **CBNV** | *** | *** | *** |  |  |  | *** | *** | *** |  |  |  |
|  | **CBNOA** | *** | *** | *** |  |  |  |  | * | * |  |  |  |  |  | **CBNO** |  |  |  |  |  |  |  |  |  |  |  |  |
|  | **CBEA** |  |  |  |  |  |  |  |  |  |  |  |  |  |  | **CBE** |  |  |  |  |  |  |  |  |  |  |  |  |
|  | **CBTA-1** | ** | ** | *** |  |  |  |  | * | * |  |  |  |  |  | **CBT-1** | *** | *** | *** |  |  |  | *** | *** | *** |  |  |  |
|  |  |  |  |  |  |  |  |  |  |  |  |  |  |  |  | **CBT-2** |  |  |  |  |  |  |  | *** | ** | * | * |  |
|  | **CBTA-3** | *** | *** | *** |  |  |  |  |  |  |  |  |  |  |  | **CBT-3** | *** | *** | *** |  |  |  | * | * | * |  |  |  |
| **(C)** | **373-12b** |  |  |  |  |  |  |  |  |  |  | * |  |  | **(F)** | **329-11b** | * | * | * |  |  |  | ** | ** | ** |  |  |  |
|  | **373-12c** |  | * | * |  |  |  |  |  | * | * | ** |  |  |  | **329-11c** |  |  |  |  |  |  |  |  |  |  |  |  |
|  | **371-14a** |  |  |  |  |  |  |  |  |  |  |  |  |  |  | **327-13a** |  |  |  |  |  |  |  |  |  |  |  |  |
|  | **417-15a** |  |  |  |  |  |  |  |  |  |  |  |  |  |  | **373-15b** |  | ** | * | * |  |  | * |  |  |  |  |  |
|  | **357-16a** |  | * |  |  |  |  |  |  |  |  |  |  |  |  | **313-16b** |  |  |  |  |  |  |  |  |  |  |  |  |
|  | **375-19a** |  |  |  |  |  |  |  |  |  |  |  |  |  |  | **331-18a** |  |  |  |  |  |  |  |  |  |  |  |  |

^a^One-way ANOVA followed by a Tukey post-hoc multiple comparisons test. A value of p ≤ 0.05 was considered significant for all tests.

**Table S6. Statistical significant differences in phytocannabinoid concentrations in Type I *Cannabis* inflorescences stored at different temperatures following one year of storage for whole versus ground samples^a^**

|  |  | **Storage Temperature [°C]** | | | |  |  |  | **Storage Temperature [°C]** | | | |
| --- | --- | --- | --- | --- | --- | --- | --- | --- | --- | --- | --- | --- |
|  |  | **-80** | **-30** | **4** | **25** |  |  |  | **-80** | **-30** | **4** | **25** |
| **(A)** | **CBGA** |  |  |  |  |  | **(D)** | **CBG** |  | * |  | * |
|  | **CBGA-C4** |  |  |  |  |  |  | **CBG-C4** |  |  |  |  |
|  | **SesquiCBGA** |  |  |  |  |  |  | **SesquiCBG** |  | * |  |  |
|  | **Δ^9^-THCA** |  |  |  |  |  |  | **Δ^9^-THC** |  |  |  |  |
|  | **Δ^9^-THCA-C4** |  |  |  |  |  |  | **Δ^9^-THC-C4** |  |  |  |  |
|  | **Δ^9^-THCVA** |  |  |  |  |  |  | **Δ^9^-THCV** |  |  |  |  |
|  | **Δ^9^-THCOA** |  |  |  |  |  |  | **Δ^9^-THCO** |  |  |  |  |
|  | **Δ^9^-THCMA** |  |  |  |  |  |  | **Δ^9^-THCM** |  |  |  |  |
|  | **CBDA** |  |  |  |  |  |  | **CBD** |  |  |  |  |
|  | **CBDA-C4** |  |  |  |  |  |  | **CBD-C4** |  |  |  |  |
|  | **CBCA** |  | * |  |  |  |  | **CBC** |  |  |  | * |
|  | **CBCVA** |  |  |  |  |  |  | **CBCV** |  |  |  |  |
|  | **CBCOA** |  |  |  |  |  |  | **CBCO** |  |  |  |  |
| **(B)** | **CBNA** |  |  |  |  |  | **(E)** | **CBN** |  |  |  | *** |
|  | **CBNA-C4** |  |  |  |  |  |  | **CBN-C4** |  |  |  |  |
|  | **CBNVA** |  |  |  |  |  |  | **CBNV** |  |  |  | *** |
|  | **CBNOA** |  |  |  |  |  |  | **CBNO** |  |  |  |  |
|  | **CBEA** |  |  |  |  |  |  | **CBE** |  |  |  |  |
|  | **CBTA-1** |  |  |  |  |  |  | **CBT-1** |  |  |  | *** |
|  |  |  |  |  |  |  |  | **CBT-2** |  |  |  | * |
|  | **CBTA-3** |  |  |  | *** |  |  | **CBT-3** |  |  |  | * |
| **(C)** | **373-12b** |  |  |  |  |  | **(F)** | **329-11b** |  |  |  | *** |
|  | **373-12c** | ** | * |  | *** |  |  | **329-11c** |  |  |  |  |
|  | **371-14a** |  |  |  |  |  |  | **327-13a** |  |  |  |  |
|  | **417-15a** |  |  |  |  |  |  | **373-15b** |  | * |  |  |
|  | **357-16a** |  |  |  |  |  |  | **313-16b** |  |  |  |  |
|  | **375-19a** |  |  |  |  |  |  | **331-18a** |  |  |  |  |

^a^One-way ANOVA followed by a Tukey post-hoc multiple comparisons test. A value of p ≤ 0.05 was considered significant for all tests.

**Table S7. Statistical significant differences in phytocannabinoid concentrations in whole and ground samples of the Type III chemovar stored at different temperatures for different storage times (t_0_ versus t_12_^a^)**

|  |  | **Whole** | | | | **Ground** | | | |  |  |  | **Whole** | | | | **Ground** | | | |
| --- | --- | --- | --- | --- | --- | --- | --- | --- | --- | --- | --- | --- | --- | --- | --- | --- | --- | --- | --- | --- |
|  |  | **Storage Temperature [°C]** | | | | **Storage Temperature [°C]** | | | |  |  |  | **Storage Temperature [°C]** | | | | **Storage Temperature [°C]** | | | |
|  |  | **-80** | **-30** | **4** | **25** | **-80** | **-30** | **4** | **25** |  |  |  | **-80** | **-30** | **4** | **25** | **-80** | **-30** | **4** | **25** |
| **(A)** | **CBGA** |  |  | * | * |  |  |  | * |  | **(D)** | **CBG** | * |  | * | * |  |  |  | * |
|  | **CBGVA** |  |  |  |  |  |  |  |  |  |  | **CBGV** |  |  |  |  |  |  |  |  |
|  | **SesquiCBGA** |  |  |  |  |  |  |  |  |  |  | **SesquiCBG** |  |  |  | * | * | * | * | * |
|  | **Δ^9^-THCA** |  |  |  |  |  |  |  | * |  |  | **Δ^9^-THC** | ** | ** | ** | *** | ** | *** | *** | *** |
|  | **Δ^9^-THCA-C4** |  |  |  | * |  |  |  | * |  |  | **Δ^9^-THC-C4** |  |  |  |  |  |  |  |  |
|  | **Δ^9^-THCVA** |  |  |  |  |  |  |  | * |  |  | **Δ^9^-THCV** |  |  |  |  |  |  |  |  |
|  | **CBDA** |  |  |  |  |  |  |  | * |  |  | **CBD** | * | * | ** | *** | * | ** | ** | *** |
|  | **CBDA-C4** |  |  | ** | *** | ** | * | ** | *** |  |  | **CBD-C4** | *** | *** | *** | *** |  |  | * | *** |
|  | **CBDVA** |  |  |  |  |  |  |  | * |  |  | **CBDV** | * | * | ** | *** |  | * | * | *** |
|  | **CBDOA** |  |  |  |  |  |  |  |  |  |  | **CBDO** |  |  |  |  |  |  |  |  |
|  | **CBDMA** |  |  |  |  |  |  |  |  |  |  | **CBDM** |  |  |  |  |  |  |  |  |
|  | **CBCA** |  |  |  |  | *** | ** | *** | *** |  |  | **CBC** |  |  |  | *** |  |  |  | *** |
|  | **CBCA-C4** | *** | *** | *** | *** | *** | *** | *** | *** |  |  | **CBC-C4** |  |  |  |  |  |  |  |  |
|  | **CBCVA** | *** | *** | *** | *** | *** | *** | *** | *** |  |  | **CBCV** | *** | *** | *** | *** | *** | *** | *** | *** |
|  | **CBCOA** | *** | *** | *** | *** | *** | *** | *** | *** |  |  | **CBCO** |  |  |  |  |  |  |  |  |
| **(B)** | **CBNA** |  |  |  |  |  |  |  |  |  | **(E)** | **CBN** | *** | *** | *** | *** |  |  |  | *** |
|  | **CBEA** | ** | * | * | *** | * | * | * | * |  |  | **CBE** |  |  |  | *** |  |  |  | *** |
|  | **CBEVA** |  |  |  | * |  |  |  |  |  |  | **CBEV** |  |  |  |  |  |  |  |  |
|  | **CBNDA** |  |  |  | *** |  |  |  | *** |  |  | **CBND** |  |  |  |  |  |  |  |  |
|  | **CBNDVA** |  |  |  |  |  |  |  |  |  |  | **CBNDV** |  |  |  |  |  |  |  |  |
|  | **CBTA-1** | * |  | * | ** | * | * | * | * |  |  | **CBT-1** |  |  |  | *** |  |  |  | *** |
|  |  |  |  |  |  |  |  |  |  |  |  | **CBT-2** | * |  |  | ** |  | * | * | ** |
|  | **CBTA-3** |  |  |  | * | * | * | * | * |  |  | **CBT-3** | * | * | * | *** |  |  |  | ** |
| **(C)** | **373-12a** | * | ** | * | *** | * | * | * | * |  | **(F)** | **329-11a** |  |  |  |  |  |  |  | ** |
|  | **373-12b** |  |  |  |  |  |  |  | ** |  |  | **329-11b** |  |  |  | *** |  |  |  | *** |
|  | **373-12c** |  |  |  |  |  |  |  |  |  |  | **329-11c** |  |  |  | *** |  | * |  | ** |
|  | **371-14a** |  |  |  |  |  |  |  |  |  |  | **327-13a** |  |  |  |  |  |  |  |  |
|  | **417-15a** |  |  |  |  |  |  |  |  |  |  | **373-15b** | * | * | * |  | * | * | * |  |
|  | **357-16a** | ** | *** | ** | * | ** | *** | ** | * |  |  | **313-16b** | ** | ** | ** | *** | * | * | * | ** |
|  | **375-19a** | * |  | * | * | * | * | * | ** |  |  | **331-18a** |  |  | * | *** |  |  |  | *** |

^a^One-way ANOVA followed by a Tukey post-hoc multiple comparisons test. A value of p ≤ 0.05 was considered significant for all tests.

Table S8. Statistical significant differences of phytocannabinoid concentrations in whole and ground samples of the Type III chemovar following one year of storage for different storage temperatures (T_1_/T_2_)

|  |  | **Whole** | | | | | | **Ground** | | | | | |  |  |  | **Whole** | | | | | | **Ground** | | | | | |
| --- | --- | --- | --- | --- | --- | --- | --- | --- | --- | --- | --- | --- | --- | --- | --- | --- | --- | --- | --- | --- | --- | --- | --- | --- | --- | --- | --- | --- |
|  |  | **Comparison of storage temperatures^a^ [°C]** | | | | | | **Comparison of storage temperatures^a^ [°C]** | | | | | |  |  |  | **Comparison of storage temperatures^a^ [°C]** | | | | | | **Comparison of storage temperatures^a^ [°C]** | | | | | |
|  |  |  |  |  |  |  |  |  |  |  |  |  |  |  |  |  |  |  |  |  |  |  |  |  |  |  |  |  |
|  |  | **25/4** | **25/**  **-30** | **25/**  **-80** | **4/**  **-30** | **4/**  **-80** | **-30/**  **-80** | **25/4** | **25/**  **-30** | **25/**  **-80** | **4/**  **-30** | **4/**  **-80** | **-30/**  **-80** |  |  |  | **25/4** | **25/**  **-30** | **25/**  **-80** | **4/**  **-30** | **4/**  **-80** | **-30/**  **-80** | **25/4** | **25/**  **-30** | **25/**  **-80** | **4/**  **-30** | **4/**  **-80** | **-30/**  **-80** |
| **(A)** | **CBGA** |  |  |  |  |  |  |  |  |  |  |  |  |  | **(D)** | **CBG** |  |  |  |  |  |  | * | * | * |  |  |  |
|  | **CBGVA** |  |  |  |  |  |  |  |  |  |  |  |  |  |  | **CBGV** |  |  |  |  |  |  |  |  |  |  |  |  |
|  | **SesquiCBGA** |  |  |  |  |  |  |  |  |  |  |  |  |  |  | **SesquiCBG** |  |  |  |  |  |  |  |  |  |  |  |  |
|  | **Δ^9^-THCA** |  |  |  |  |  |  |  | *** |  |  |  |  |  |  | **Δ^9^-THC** | *** | *** | *** |  |  |  | *** | *** | *** |  |  |  |
|  | **Δ^9^-THCA-C4** |  |  |  |  |  |  |  |  |  |  |  |  |  |  | **Δ^9^-THC-C4** |  |  |  |  |  |  |  |  |  |  |  |  |
|  | **Δ^9^-THCVA** |  |  |  |  |  |  |  |  |  |  |  |  |  |  | **Δ^9^-THCV** |  |  |  |  |  |  |  |  |  |  |  |  |
|  | **CBDA** |  |  |  |  |  |  |  |  |  |  |  |  |  |  | **CBD** | *** | *** | *** |  |  |  | *** | *** | *** |  |  |  |
|  | **CBDA-C4** | * | *** | *** | * | ** |  |  | * |  |  |  |  |  |  | **CBD-C4** | *** | *** | *** |  |  |  | ** | *** | *** |  |  |  |
|  | **CBDVA** |  |  |  |  |  |  |  |  |  |  |  |  |  |  | **CBDV** | *** | *** | *** |  |  |  | *** | *** | *** |  |  |  |
|  | **CBDOA** |  |  |  |  |  |  |  |  |  |  |  |  |  |  | **CBDO** |  |  |  |  |  |  |  |  |  |  |  |  |
|  | **CBDMA** |  |  |  |  |  |  |  |  |  |  |  |  |  |  | **CBDM** |  |  |  |  |  |  |  |  |  |  |  |  |
|  | **CBCA** |  |  |  |  |  |  |  | * |  |  |  |  |  |  | **CBC** | * | ** | ** |  |  |  | ** | ** | ** |  |  |  |
|  | **CBCA-C4** |  |  |  |  |  |  |  | * |  |  |  |  |  |  | **CBC-C4** |  |  |  |  |  |  |  |  |  |  |  |  |
|  | **CBCVA** |  |  |  |  |  |  |  |  |  |  |  |  |  |  | **CBCV** | *** | *** | *** | * | * |  | *** | *** | *** |  |  |  |
|  | **CBCOA** |  |  |  |  |  |  |  |  |  |  |  |  |  |  | **CBCO** |  |  |  |  |  |  |  |  |  |  |  |  |
| **(B)** | **CBNA** |  |  |  |  |  |  |  |  |  |  |  |  |  | **(E)** | **CBN** | ** | *** | *** |  |  |  | * | ** | ** |  |  |  |
|  | **CBEA** | * | * |  |  |  |  |  |  |  |  |  |  |  |  | **CBE** | *** | *** | *** |  |  |  | * | ** | *** |  |  |  |
|  | **CBEVA** |  |  |  |  |  |  |  |  |  |  |  |  |  |  | **CBEV** |  |  |  |  |  |  |  |  |  |  |  |  |
|  | **CBNDA** | *** | *** | *** |  |  |  | *** | *** | *** |  |  |  |  |  | **CBND** |  |  |  |  |  |  |  |  |  |  |  |  |
|  | **CBNDVA** |  |  |  |  |  |  |  |  |  |  |  |  |  |  | **CBNDV** |  |  |  |  |  |  |  |  |  |  |  |  |
|  | **CBTA-1** |  |  |  |  |  |  |  |  |  |  |  |  |  |  | **CBT-1** | *** | *** | *** |  |  |  | *** | *** | *** |  |  |  |
|  |  |  |  |  |  |  |  |  |  |  |  |  |  |  |  | **CBT-2** | * | * | * |  |  |  | * |  | * |  |  |  |
|  | **CBTA-3** |  |  |  |  |  |  |  |  |  |  |  |  |  |  | **CBT-3** | ** | *** | ** |  |  |  | * | * | * |  |  |  |
| **(C)** | **373-12a** |  |  | * |  |  |  |  |  |  |  |  |  |  | **(F)** | **329-11a** |  |  |  |  |  |  | ** | ** | ** |  |  |  |
|  | **373-12b** |  |  |  |  |  |  | * | * | * |  |  |  |  |  | **329-11b** | *** | *** | *** |  |  |  | ** | *** | *** |  |  |  |
|  | **373-12c** |  |  |  |  |  |  |  |  |  |  |  |  |  |  | **329-11c** | * | * | * |  |  |  | * | * | * |  |  |  |
|  | **371-14a** |  |  |  |  |  |  |  |  |  |  |  |  |  |  | **327-13a** |  |  |  |  |  |  |  |  |  |  |  |  |
|  | **417-15a** |  |  |  |  |  |  |  |  |  |  |  |  |  |  | **373-15b** |  |  |  |  |  |  | * | * | * |  |  |  |
|  | **357-16a** |  | *** |  | ** |  |  | * | * |  |  |  |  |  |  | **313-16b** |  |  |  |  |  |  |  |  |  |  |  |  |
|  | **375-19a** |  |  |  |  |  |  |  |  |  |  |  |  |  |  | **331-18a** | *** | *** | *** |  |  |  | *** | *** | *** |  |  |  |

^a^One-way ANOVA followed by a Tukey post-hoc multiple comparisons test. A value of p ≤ 0.05 was considered significant for all tests.

**Table S9. Statistical significant differences in phytocannabinoid concentrations in Type III *Cannabis* inflorescences stored at different temperatures for one year for whole versus ground samples^a^**

|  |  | **Storage Temperature [°C]** | | | |  |  |  | **Storage Temperature [°C]** | | | |
| --- | --- | --- | --- | --- | --- | --- | --- | --- | --- | --- | --- | --- |
|  |  | **-80** | **-30** | **4** | **25** |  |  |  | **-80** | **-30** | **4** | **25** |
| **(A)** | **CBGA** |  |  |  |  |  | **(D)** | **CBG** |  |  |  |  |
|  | **CBGVA** |  |  |  |  |  |  | **CBGV** |  |  |  |  |
|  | **SesquiCBGA** |  |  |  |  |  |  | **SesquiCBG** |  |  |  |  |
|  | **Δ^9^-THCA** |  |  |  |  |  |  | **Δ^9^-THC** |  |  |  | * |
|  | **Δ^9^-THCA-C4** |  |  |  |  |  |  | **Δ^9^-THC-C4** |  |  |  |  |
|  | **Δ^9^-THCVA** |  |  |  |  |  |  | **Δ^9^-THCV** |  |  |  |  |
|  | **CBDA** |  |  |  |  |  |  | **CBD** |  |  |  | * |
|  | **CBDA-C4** |  |  |  |  |  |  | **CBD-C4** |  |  |  | ** |
|  | **CBDVA** |  |  |  |  |  |  | **CBDV** |  |  |  |  |
|  | **CBDOA** |  |  |  |  |  |  | **CBDO** |  |  |  |  |
|  | **CBDMA** |  |  |  |  |  |  | **CBDM** |  |  |  |  |
|  | **CBCA** |  |  |  |  |  |  | **CBC** |  |  |  |  |
|  | **CBCA-C4** |  |  |  |  |  |  | **CBC-C4** |  |  |  |  |
|  | **CBCVA** |  |  |  |  |  |  | **CBCV** |  |  |  | * |
|  | **CBCOA** |  |  |  | * |  |  | **CBCO** |  |  |  |  |
| **(B)** | **CBNA** |  |  |  |  |  | **(E)** | **CBN** |  |  |  |  |
|  | **CBEA** |  |  |  |  |  |  | **CBE** |  |  |  |  |
|  | **CBEVA** |  |  |  |  |  |  | **CBEV** |  |  |  |  |
|  | **CBNDA** |  |  |  | * |  |  | **CBND** |  |  |  |  |
|  | **CBNDVA** |  |  |  |  |  |  | **CBNDV** |  |  |  |  |
|  | **CBTA-1** |  |  |  |  |  |  | **CBT-1** |  |  |  |  |
|  |  |  |  |  |  |  |  | **CBT-2** |  |  |  |  |
|  | **CBTA-3** |  |  |  |  |  |  | **CBT-3** |  |  |  |  |
| **(C)** | **373-12a** |  |  |  | * |  | **(F)** | **329-11a** |  |  |  | * |
|  | **373-12b** |  |  |  |  |  |  | **329-11b** |  |  |  | * |
|  | **373-12c** |  |  |  |  |  |  | **329-11c** |  |  |  |  |
|  | **371-14a** |  |  |  |  |  |  | **327-13a** |  |  |  |  |
|  | **417-15a** |  |  |  |  |  |  | **373-15b** |  |  |  |  |
|  | **357-16a** |  |  |  |  |  |  | **313-16b** |  |  |  |  |
|  | **375-19a** |  |  |  |  |  |  | **331-18a** |  |  |  | ** |

^a^One-way ANOVA followed by a Tukey post-hoc multiple comparisons test. A value of p ≤ 0.05 was considered significant for all tests.

**Table S10. Statistical significant differences in the concentration of major neutral phytocannabinoid (CNB) in extracts dissolved in DMSO, ethanol, and olive oil at different storage times for different storage temperatures (T_1_/T_2_)**

|  |  |  | **DMSO** | | | | | | **Ethanol** | | | | | | **Olive Oil** | | | | | |
| --- | --- | --- | --- | --- | --- | --- | --- | --- | --- | --- | --- | --- | --- | --- | --- | --- | --- | --- | --- | --- |
| **Time** | **Chemovar Type** | **CNB** | **Comparison of storage temperatures^a^ [°C]** | | | | | | **Comparison of storage temperatures^a^ [°C]** | | | | | | **Comparison of storage temperatures^a^ [°C]** | | | | | |
|  |  |  | **25/**  **4** | **25/**  **-30** | **25/**  **-80** | **4/**  **-30** | **4/**  **-80** | **-30/**  **-80** | **25/**  **4** | **25/**  **-30** | **25/**  **-80** | **4/**  **-30** | **4/**  **-80** | **-30/**  **-80** | **25/**  **4** | **25/**  **-30** | **25/**  **-80** | **4/**  **-30** | **4/**  **-80** | **-30/**  **-80** |
| **t_6_** | I | Δ^9^-THC | *** | *** | *** | *** | ** |  | *** | *** | *** | * | * |  | *** |  | * | *** | *** | * |
|  |  | CBN | *** | *** | *** | *** | * | * | *** | *** | *** |  |  |  | ** | * | * | * | * |  |
|  |  | CBD | *** | *** | *** | * |  | * | *** | *** | *** |  |  |  |  |  |  |  |  |  |
|  | III | Δ^9^-THC | *** | *** | *** |  |  |  | *** | *** | *** |  |  |  | *** | *** | *** | ** | *** |  |
|  |  | CBN | *** | *** | *** | *** | *** |  | *** | *** | *** |  |  |  | *** | * | * | * | * |  |
|  |  | CBD | *** | *** | *** | * | * |  | *** | *** | *** | * |  |  |  | *** | *** | *** | *** |  |
| **t_12_** | I | Δ^9^-THC | *** | *** | *** | *** | *** |  | *** | *** | *** | *** | *** |  | *** | *** | *** | *** | ** |  |
|  |  | CBN | *** | *** | *** | *** | *** |  | *** | *** | *** | * |  |  | *** | *** | *** |  |  |  |
|  |  | CBD | *** | *** | *** | *** | *** |  | *** | *** | *** |  |  |  |  |  |  |  |  |  |
|  | III | Δ^9^-THC | *** | *** | *** | ** | ** |  | *** | *** | *** | ** | * |  | *** | *** | *** |  |  |  |
|  |  | CBN | *** | *** | *** | *** | *** |  | *** | *** | *** | *** | *** |  | ** | ** | ** |  |  |  |
|  |  | CBD | *** | *** | *** | ** | ** |  | *** | *** | *** | ** | ** |  | *** | *** | *** | * | * |  |

^a^One-way ANOVA followed by a Tukey post-hoc multiple comparisons test. A value of p ≤ 0.05 was considered significant for all tests.

**Table S11. Statistical significant differences in the concentration of major neutral phytocannabinoid (CNB) in *Cannabis* extracts over time for different storage solvents**

|  |  |  | **DMSO vs. Ethanol^a^** | | | | **DMSO vs. Olive Oil^a^** | | | | **Ethanol vs. Olive Oil^a^** | | | |
| --- | --- | --- | --- | --- | --- | --- | --- | --- | --- | --- | --- | --- | --- | --- |
| **Time** | **Chemovar Type** | **CNB** | **Storage Temperature [°C]** | | | | **Storage Temperature [°C]** | | | | **Storage Temperature [°C]** | | | |
|  |  |  | **-80** | **-30** | **4** | **25** | **-80** | **-30** | **4** | **25** | **-80** | **-30** | **4** | **25** |
| **t_6_** | I | Δ^9^-THC | *** | *** | *** | *** | *** | *** | *** | *** |  |  | *** | *** |
|  |  | CBN | *** | *** | *** | *** | *** | *** | *** | *** | *** | *** |  | *** |
|  |  | CBD | *** | *** | *** | *** | *** | *** | *** | *** |  |  |  | *** |
|  | III | Δ^9^-THC | * | * | * |  |  | * | *** | *** |  |  | *** | *** |
|  |  | CBN | *** | *** | *** | *** | *** | *** | *** | *** |  |  | *** | *** |
|  |  | CBD | *** | *** | *** | *** | *** | *** | *** | *** |  |  | *** | *** |
| **t_12_** | I | Δ^9^-THC |  |  |  |  |  |  |  |  |  |  |  |  |
|  |  | CBN | *** | *** | *** | *** | *** | *** | *** | *** |  |  |  | *** |
|  |  | CBD | *** | *** | *** | *** | *** | *** | *** | *** |  |  |  | *** |
|  | III | Δ^9^-THC | ** | ** | *** | *** | *** | ** | *** | *** |  |  | * | *** |
|  |  | CBN | *** | ** | *** | *** | *** | ** | *** | *** |  |  |  | * |
|  |  | CBD | *** | *** | *** | *** | *** | *** | *** | *** |  |  |  | *** |

^a^Two-way ANOVA followed by a Sidak post-hoc multiple comparisons test. A value of p ≤ 0.05 was considered significant for all tests.

**Table S12. Statistical significant differences in phytocannabinoid concentrations in Type I extracts dissolved in DMSO, ethanol or olive oil for different storage times (t_0_ versus t_12_^a^)**

|  |  | DMSO | | | | Ethanol | | | | Olive Oil | | | |  |  | DMSO | | | | Ethanol | | | | Olive Oil | | | |
| --- | --- | --- | --- | --- | --- | --- | --- | --- | --- | --- | --- | --- | --- | --- | --- | --- | --- | --- | --- | --- | --- | --- | --- | --- | --- | --- | --- |
|  |  | **Storage Temperature [°C]** | | | | **Storage Temperature [°C]** | | | | **Storage Temperature [°C]** | | | |  |  | **Storage Temperature [°C]** | | | | **Storage Temperature [°C]** | | | | **Storage Temperature [°C]** | | | |
|  |  | **-80** | **-30** | **4** | **25** | **-80** | **-30** | **4** | **25** | **-80** | **-30** | **4** | **25** |  |  | **-80** | **-30** | **4** | **25** | **-80** | **-30** | **4** | **25** | **-80** | **-30** | **4** | **25** |
| **(A)** | **CBGA** | *** | *** | *** | *** | * | * | * | *** | ** | ** | * | ** | **(D)** | **CBG** | ** | ** | ** | *** |  |  |  | *** |  |  |  |  |
|  | **CBGA-C4** | *** | *** | ** | *** | *** | *** | *** | *** | *** | *** | *** | *** |  | **CBG-C4** |  |  |  |  |  |  |  | *** |  |  |  |  |
|  | **SesquiCBGA** |  |  |  | ** | * | * |  |  | * | * | * | * |  | **SesquiCBG** | *** | *** | *** | *** | *** | *** | *** | *** | *** | *** | *** | *** |
|  | **Δ^9^-THCA** |  |  |  | *** | * |  |  | *** |  |  |  |  |  | **Δ^9^-THC** |  | * | * | *** |  |  |  | *** |  |  |  | ** |
|  | **Δ^9^-THCA-C4** |  |  |  | *** |  |  |  | *** |  |  |  |  |  | **Δ^9^-THC-C4** | * | * | * | *** |  |  |  | *** |  |  |  |  |
|  | **Δ^9^-THCVA** | ** | ** | *** | *** | ** | ** | ** | *** | *** | *** | *** | *** |  | **Δ^9^-THCV** | *** | *** | *** | *** | *** | *** | *** | *** | *** | *** | *** | *** |
|  | **Δ^9^-THCOA** |  |  |  |  |  |  |  |  |  |  |  |  |  | **Δ^9^-THCO** |  |  |  |  |  |  |  | *** |  |  |  |  |
|  | **Δ^9^-THCMA** | * | * |  |  |  |  |  |  |  |  |  |  |  | **Δ^9^-THCM** |  |  |  |  |  |  |  |  |  |  |  |  |
|  | **CBDA** | * | ** | *** | *** |  |  |  | *** |  |  |  |  |  | **CBD** | ** | *** | ** | *** |  |  | * | *** |  |  |  |  |
|  | **CBDA-C4** |  |  |  |  | * | * | * | *** |  |  |  |  |  | **CBD-C4** |  |  |  |  |  |  |  |  |  |  |  |  |
|  | **CBCA** | * | * | *** | *** | *** | *** | *** | *** | *** | *** | *** | *** |  | **CBC** |  |  | * |  |  |  |  | *** | ** | ** | *** | ** |
|  | **CBCVA** |  |  |  |  |  |  |  |  |  |  |  |  |  | **CBCV** |  |  |  |  |  |  |  |  |  |  |  |  |
|  | **CBCOA** |  |  |  |  |  |  |  |  |  |  |  |  |  | **CBCO** |  |  |  |  |  |  |  |  |  |  |  |  |
| **(B)** | **CBNA** |  |  |  |  | * | * | * | ** | * | * | * |  | **(E)** | **CBN** |  |  |  | *** | * | * | * | *** | * | * | * |  |
|  | **CBNA-C4** | *** | ** | * |  |  |  |  |  |  |  | * | * |  | **CBN-C4** |  |  |  | *** |  |  |  |  |  |  |  |  |
|  | **CBNVA** |  |  |  |  |  |  |  |  |  |  |  | * |  | **CBNV** | *** | *** | *** | *** | * |  | * | *** |  |  |  | *** |
|  | **CBNOA** |  |  |  |  | *** | *** | *** | ** |  |  |  | * |  | **CBNO** |  |  |  | *** |  |  |  |  |  |  |  |  |
|  | **CBEA** |  |  |  |  | *** | *** | *** | * |  |  |  |  |  | **CBE** |  |  |  |  |  |  |  |  |  |  |  |  |
|  | **CBTA-1** |  |  |  |  |  |  |  |  |  |  |  |  |  | **CBT-1** | *** | *** | *** |  |  |  |  | *** | * | * | * | *** |
|  |  |  |  |  |  |  |  |  |  |  |  |  |  |  | **CBT-2** |  |  |  |  |  |  |  | *** |  |  |  |  |
|  | **CBTA-3** | *** | *** | *** | *** | ** | ** | ** | *** | * |  | * | ** |  | **CBT-3** |  |  | * | *** |  |  |  |  |  |  |  |  |
| **(C)** | **373-12b** |  |  |  | * |  |  |  | * |  |  |  |  | **(F)** | **329-11b** | ** | ** | ** | *** |  |  |  | *** |  |  |  | * |
|  | **373-12c** | *** | *** | *** | *** | *** | *** | *** | *** | *** | *** | *** | *** |  | **329-11c** |  |  |  |  |  |  |  | *** |  |  |  |  |
|  | **371-14a** |  |  |  |  |  |  |  |  |  |  |  |  |  | **327-13a** |  |  |  |  |  |  |  |  |  |  |  |  |
|  | **417-15a** |  |  |  |  |  | * | *** | *** |  |  |  |  |  | **373-15b** | * | * | *** | *** | ** | ** | ** | *** | *** | *** | *** | *** |
|  | **357-16a** | *** | *** | *** | *** | *** | *** | *** | *** | *** | *** | *** | *** |  | **313-16b** | * | * | * | ** | * | * | * | *** |  |  |  |  |
|  | **375-19a** |  |  |  |  |  |  |  |  |  |  |  |  |  | **331-18a** | * | * |  | *** |  |  |  | *** |  |  |  |  |

^a^One-way ANOVA followed by a Tukey post-hoc multiple comparisons test. A value of p ≤ 0.05 was considered significant for all tests.

**Table S13i. Statistical significant differences in phytocannabinoid concentrations in Type I extracts dissolved in DMSO and stored for one year for different storage temperatures (T_1_/T_2_)**

|  |  | **DMSO** | | | | | | | | | | | | | |
| --- | --- | --- | --- | --- | --- | --- | --- | --- | --- | --- | --- | --- | --- | --- | --- |
|  |  | **Comparison of storage temperatures^a^ [°C]** | | | | | |  |  | **Comparison of storage temperatures^a^ [°C]** | | | | | |
|  |  | **25/4** | **25/-30** | **25/-80** | **4/-30** | **4/-80** | **-30/-80** |  |  | **25/4** | **25/-30** | **25/-80** | **4/-30** | **4/-80** | **-30/-80** |
| **(A)** | **CBGA** | *** | *** | *** | *** | ** | * | **(D)** | **CBG** | *** | *** | *** |  |  |  |
|  | **CBGA-C4** |  | ** |  | ** |  | * |  | **CBG-C4** |  |  |  |  |  |  |
|  | **SesquiCBGA** | *** | *** | *** | ** | ** |  |  | **SesquiCBG** | * |  | * | * | * |  |
|  | **Δ^9^-THCA** | *** | *** | *** |  |  |  |  | **Δ^9^-THC** | *** | *** | *** |  |  |  |
|  | **Δ^9^-THCA-C4** | *** | *** | *** | ** | ** |  |  | **Δ^9^-THC-C4** | *** | *** | *** |  |  |  |
|  | **Δ^9^-THCVA** | *** | *** | *** | *** | *** |  |  | **Δ^9^-THCV** | *** | *** | *** |  |  |  |
|  | **Δ^9^-THCOA** | *** | *** | *** | *** | ** |  |  | **Δ^9^-THCO** |  |  |  |  |  |  |
|  | **Δ^9^-THCMA** | *** | *** | *** | ** | *** |  |  | **Δ^9^-THCM** |  |  |  |  |  |  |
|  | **CBDA** | *** | *** | *** | *** | *** |  |  | **CBD** | *** | *** | *** |  |  |  |
|  | **CBDA-C4** |  |  |  |  |  |  |  | **CBD-C4** |  |  |  |  |  |  |
|  | **CBCA** | ** | *** | *** | * | * |  |  | **CBC** | *** | *** | ** | * | ** |  |
|  | **CBCVA** |  | * | * | * |  |  |  | **CBCV** |  |  |  |  |  |  |
|  | **CBCOA** |  |  |  |  |  |  |  | **CBCO** |  |  |  |  |  |  |
| **(B)** | **CBNA** | * | *** | *** | ** | ** |  | **(E)** | **CBN** | *** | *** | *** |  | * |  |
|  | **CBNA-C4** |  | * | *** | * | *** | * |  | **CBN-C4** | *** | *** | *** |  |  |  |
|  | **CBNVA** |  |  |  |  |  |  |  | **CBNV** | *** | *** | *** |  |  |  |
|  | **CBNOA** |  |  |  |  |  |  |  | **CBNO** | *** | *** | *** |  |  |  |
|  | **CBTA-1** |  |  |  |  |  |  |  | **CBT-1** | *** | *** | *** | *** | *** |  |
|  |  |  |  |  |  |  |  |  | **CBT-2** |  |  |  |  |  |  |
|  | **CBTA-3** |  | * |  |  | * | ** |  | **CBT-3** | *** | *** | *** |  |  |  |
| **(C)** | **373-12b** | * | *** | *** | * | * |  | **(F)** | **329-11b** | *** | *** | *** |  |  |  |
|  | **373-12c** |  |  |  |  |  |  |  | **329-11c** |  |  |  |  |  |  |
|  | **371-14a** |  |  |  |  |  |  |  | **327-13a** |  |  |  |  |  |  |
|  | **417-15a** |  |  |  |  |  |  |  | **373-15b** | *** | *** | *** | *** | *** | * |
|  | **357-16a** | *** | *** | *** | * | * |  |  | **313-16b** | * | * |  |  | * |  |
|  | **375-19a** | *** | *** | *** | * | ** |  |  | **331-18a** | *** | *** | *** |  |  |  |

^a^One-way ANOVA followed by a Tukey post-hoc multiple comparisons test. A value of p ≤ 0.05 was considered significant for all tests.

**Table S13ii. Statistical significant differences in phytocannabinoid concentrations in Type I extracts dissolved in ethanol and stored for one year for different storage temperatures (T_1_/T_2_)**

|  |  | **Ethanol** | | | | | | | | | | | | | |
| --- | --- | --- | --- | --- | --- | --- | --- | --- | --- | --- | --- | --- | --- | --- | --- |
|  |  | **Comparison of storage temperatures^a^ [°C]** | | | | | |  |  | **Comparison of storage temperatures^a^ [°C]** | | | | | |
|  |  | **25/4** | **25/-30** | **25/-80** | **4/-30** | **4/-80** | **-30/-80** |  |  | **25/4** | **25/-30** | **25/-80** | **4/-30** | **4/-80** | **-30/-80** |
| **(A)** | **CBGA** | *** | *** | *** |  |  |  | **(D)** | **CBG** | *** | *** | *** | * | * |  |
|  | **CBGA-C4** | *** | *** | *** |  |  |  |  | **CBG-C4** | *** | *** | *** |  |  |  |
|  | **SesquiCBGA** | *** | *** | *** |  | * |  |  | **SesquiCBG** | * | *** | ** |  |  |  |
|  | **Δ^9^-THCA** | *** | *** | *** |  |  |  |  | **Δ^9^-THC** | *** | *** | *** | * | * |  |
|  | **Δ^9^-THCA-C4** | *** | *** | *** |  | * |  |  | **Δ^9^-THC-C4** | *** | *** | *** | * | * |  |
|  | **Δ^9^-THCVA** | *** | *** | *** |  | * |  |  | **Δ^9^-THCV** | *** | *** | *** | * | * |  |
|  | **Δ^9^-THCOA** | *** | *** | *** |  |  |  |  | **Δ^9^-THCO** | *** | *** | *** |  |  |  |
|  | **Δ^9^-THCMA** | *** | *** | *** | * | * |  |  | **Δ^9^-THCM** |  |  |  |  |  |  |
|  | **CBDA** | *** | *** | *** |  |  |  |  | **CBD** | *** | *** | *** | * | * |  |
|  | **CBDA-C4** | *** | *** | *** |  |  |  |  | **CBD-C4** |  |  |  |  |  |  |
|  | **CBCA** | *** | *** | *** |  |  |  |  | **CBC** | *** | *** | *** |  |  |  |
|  | **CBCVA** |  |  |  |  |  |  |  | **CBCV** |  |  |  |  |  |  |
|  | **CBCOA** |  |  |  |  |  |  |  | **CBCO** |  |  |  |  |  |  |
| **(B)** | **CBNA** | *** | *** | *** |  |  |  | **(E)** | **CBN** | *** | *** | *** |  |  |  |
|  | **CBNA-C4** | *** | *** | *** |  | * |  |  | **CBN-C4** |  |  |  |  |  |  |
|  | **CBNVA** | *** | *** | *** |  |  |  |  | **CBNV** | *** | *** | *** |  |  |  |
|  | **CBNOA** | * | * | ** |  |  |  |  | **CBNO** |  |  |  |  |  |  |
|  | **CBTA-1** | *** | *** | *** |  |  |  |  | **CBT-1** | *** | *** | *** |  |  |  |
|  |  |  |  |  |  |  |  |  | **CBT-2** | *** | *** | *** |  |  |  |
|  | **CBTA-3** |  | *** | *** | ** | ** |  |  | **CBT-3** | *** | *** | *** | * | * |  |
| **(C)** | **373-12b** | *** | *** | *** |  |  |  | **(F)** | **329-11b** | *** | *** | *** | * | * |  |
|  | **373-12c** | * | ** | ** |  |  |  |  | **329-11c** | *** | *** | *** |  |  |  |
|  | **371-14a** |  |  |  |  |  |  |  | **327-13a** |  |  |  |  |  |  |
|  | **417-15a** | *** | *** | *** | *** | *** |  |  | **373-15b** | *** | *** | *** |  |  |  |
|  | **357-16a** | *** | *** | *** | * | * |  |  | **313-16b** | *** | *** | *** |  |  |  |
|  | **375-19a** | *** | *** | *** |  |  |  |  | **331-18a** | *** | *** | *** |  |  |  |

^a^One-way ANOVA followed by a Tukey post-hoc multiple comparisons test. A value of p ≤ 0.05 was considered significant for all tests.

**Table S13iii. Statistical significant differences in phytocannabinoid concentrations in Type I extracts dissolved in olive oil and stored for one year for different storage temperatures (T_1_/T_2_)**

|  |  | **Olive Oil** | | | | | | | | | | | | | |
| --- | --- | --- | --- | --- | --- | --- | --- | --- | --- | --- | --- | --- | --- | --- | --- |
|  |  | **Comparison of storage temperatures^a^ [°C]** | | | | | |  |  | **Comparison of storage temperatures^a^ [°C]** | | | | | |
|  |  | **25/4** | **25/-30** | **25/-80** | **4/-30** | **4/-80** | **-30/-80** |  |  | **25/4** | **25/-30** | **25/-80** | **4/-30** | **4/-80** | **-30/-80** |
| **(A)** | **CBGA** |  |  |  |  |  |  | **(D)** | **CBG** |  |  |  |  |  |  |
|  | **CBGA-C4** |  |  |  |  |  |  |  | **CBG-C4** |  |  |  |  |  |  |
|  | **SesquiCBGA** | * | * |  |  |  |  |  | **SesquiCBG** | *** | ** | ** | * | * |  |
|  | **Δ^9^-THCA** |  |  | * |  |  |  |  | **Δ^9^-THC** | *** | *** | *** |  |  |  |
|  | **Δ^9^-THCA-C4** | * | * |  |  |  |  |  | **Δ^9^-THC-C4** |  |  |  |  |  |  |
|  | **Δ^9^-THCVA** | * | * | * |  |  |  |  | **Δ^9^-THCV** | * | ** | ** | * | * |  |
|  | **Δ^9^-THCOA** | * |  |  |  |  |  |  | **Δ^9^-THCO** |  |  |  |  |  |  |
|  | **Δ^9^-THCMA** | * | * | * |  |  |  |  | **Δ^9^-THCM** |  |  |  |  |  |  |
|  | **CBDA** |  |  |  | * |  |  |  | **CBD** |  |  |  |  |  |  |
|  | **CBDA-C4** |  |  |  |  |  |  |  | **CBD-C4** |  |  |  |  |  |  |
|  | **CBCA** |  |  | * |  |  | * |  | **CBC** |  |  |  | * |  |  |
|  | **CBCVA** |  |  |  |  |  |  |  | **CBCV** |  |  |  |  |  |  |
|  | **CBCOA** |  |  |  |  |  |  |  | **CBCO** |  |  |  |  |  |  |
| **(B)** | **CBNA** | *** | *** | *** |  | * |  | **(E)** | **CBN** | *** | *** | *** | * | *** |  |
|  | **CBNA-C4** | *** | *** | *** |  |  |  |  | **CBN-C4** |  |  |  |  |  |  |
|  | **CBNVA** | *** | *** | *** |  |  |  |  | **CBNV** | *** | *** | *** |  |  |  |
|  | **CBNOA** | * | * | * |  |  |  |  | **CBNO** |  |  |  |  |  |  |
|  | **CBTA-1** | * |  |  | * | * |  |  | **CBT-1** | *** | *** | *** |  | * |  |
|  |  |  |  |  |  |  |  |  | **CBT-2** |  |  |  |  |  |  |
|  | **CBTA-3** | *** | *** | *** |  |  |  |  | **CBT-3** | *** | *** | *** |  |  |  |
| **(C)** | **373-12b** | * | * | * |  |  |  | **(F)** | **329-11b** | *** | *** | *** |  |  |  |
|  | **373-12c** |  |  |  |  |  |  |  | **329-11c** |  |  |  |  |  |  |
|  | **371-14a** |  |  |  |  |  |  |  | **327-13a** |  |  |  |  |  |  |
|  | **417-15a** |  | ** | *** | * | *** | * |  | **373-15b** | * |  |  | * | * |  |
|  | **357-16a** |  |  |  |  |  |  |  | **313-16b** |  |  |  |  |  |  |
|  | **375-19a** |  |  |  |  |  |  |  | **331-18a** |  |  |  |  |  |  |

^a^One-way ANOVA followed by a Tukey post-hoc multiple comparisons test. A value of p ≤ 0.05 was considered significant for all tests.

**Table S14. Statistical significant differences in phytocannabinoid concentrations in Type I *Cannabis* extracts following one year for different storage solvents**

|  |  | **DMSO vs. Ethanol^a^** | | | | **DMSO vs. Olive Oil^a^** | | | | **Ethanol vs. Olive Oil^a^** | | | |  |  | **DMSO vs. Ethanol^a^** | | | | **DMSO vs. Olive Oil^a^** | | | | **Ethanol vs. Olive Oil^a^** | | | |
| --- | --- | --- | --- | --- | --- | --- | --- | --- | --- | --- | --- | --- | --- | --- | --- | --- | --- | --- | --- | --- | --- | --- | --- | --- | --- | --- | --- |
|  |  | **Storage Temperature [°C]** | | | | **Storage Temperature [°C]** | | | | **Storage Temperature [°C]** | | | |  |  | **Storage Temperature [°C]** | | | | **Storage Temperature [°C]** | | | | **Storage Temperature [°C]** | | | |
|  |  | **-80** | **-30** | **4** | **25** | **-80** | **-30** | **4** | **25** | **-80** | **-30** | **4** | **25** |  |  | **-80** | **-30** | **4** | **25** | **-80** | **-30** | **4** | **25** | **-80** | **-30** | **4** | **25** |
| **(A)** | **CBGA** | ** | * | *** | *** | *** | ** | *** | *** |  |  | * | *** | **(D)** | **CBG** | *** | *** | *** | *** | *** | *** | *** | *** |  |  |  | *** |
|  | **CBGA-C4** | ** | *** | * |  |  | *** |  |  |  | * | ** |  |  | **CBG-C4** |  |  |  | *** |  |  |  |  |  |  |  | *** |
|  | **SesquiCBGA** | *** | *** | *** | *** | *** | *** | *** | *** |  |  | *** | *** |  | **SesquiCBG** | *** | *** |  |  | *** | *** |  | *** |  | * |  | *** |
|  | **Δ^9^-THCA** |  |  |  |  |  |  |  | *** |  |  |  | *** |  | **Δ^9^-THC** | * | * | * |  | * | ** | ** | *** |  |  |  | *** |
|  | **Δ^9^-THCA-C4** | *** | * | *** |  |  | ** |  | *** | *** | *** | *** | *** |  | **Δ^9^-THC-C4** | *** | *** | *** | *** | *** | *** | *** | *** | ** | ** | *** | *** |
|  | **Δ^9^-THCVA** |  |  | *** |  | *** | *** |  | *** | *** | *** | *** | *** |  | **Δ^9^-THCV** |  | * | * | *** | *** | *** | * | *** | *** | *** | *** | *** |
|  | **Δ^9^-THCOA** | * | *** |  | *** | *** | *** | ** | *** | *** | *** | *** | *** |  | **Δ^9^-THCO** |  |  |  | *** |  |  |  |  |  |  |  | *** |
|  | **Δ^9^-THCMA** | *** | *** | *** |  | *** | *** | *** | *** |  |  |  | *** |  | **Δ^9^-THCM** |  |  |  |  |  |  |  |  |  |  |  |  |
|  | **CBDA** |  |  | *** | * | ** |  | *** | *** |  |  | ** | *** |  | **CBD** | *** | *** | *** | *** | *** | *** | *** | *** |  |  |  | *** |
|  | **CBDA-C4** |  | * | * | *** | * |  |  |  | * | * | * | *** |  | **CBD-C4** |  |  |  |  |  |  |  |  |  |  |  |  |
|  | **CBCA** | *** | *** | *** | *** | *** | *** | ** | * |  | ** | ** | *** |  | **CBC** |  |  |  | *** | *** | ** | ** | *** | ** |  | *** | *** |
|  | **CBCVA** | *** | *** | *** | *** | *** | *** | *** | *** |  |  |  |  |  | **CBCV** |  |  |  |  |  |  |  |  |  |  |  |  |
|  | **CBCOA** |  |  |  |  |  |  |  |  |  |  |  |  |  | **CBCO** |  |  |  |  |  |  |  |  |  |  |  |  |
| **(B)** | **CBNA** | *** | *** | *** | *** | *** | *** | *** | *** | *** | *** | *** | *** | **(E)** | **CBN** | *** | *** | * | *** | *** | *** |  | *** |  |  |  | *** |
|  | **CBNA-C4** | *** | *** | ** | *** | *** | *** |  | ** |  |  | * | *** |  | **CBN-C4** |  |  |  | *** |  |  |  | *** |  |  |  | *** |
|  | **CBNVA** |  | * |  |  |  |  | * |  |  |  |  |  |  | **CBNV** | *** | *** | *** | *** | *** | *** | *** | *** |  |  |  | *** |
|  | **CBNOA** | * | * | * |  |  |  |  | *** |  |  | * | * |  | **CBNO** |  |  |  | *** |  |  |  | *** |  |  |  | *** |
|  | **CBEA** |  |  |  |  | * |  |  |  |  |  |  |  |  | **CBE** | *** | *** | *** | ** | *** | *** | *** |  | *** | *** | *** | ** |
|  | **CBTA-1** | *** | *** | *** |  | *** | *** | * | *** |  |  |  | *** |  | **CBT-1** | *** | *** | *** | *** | *** | *** | *** | *** |  | * | * | ** |
|  |  |  |  |  |  |  |  |  |  |  |  |  |  |  | **CBT-2** |  |  | * | *** | * |  |  |  |  |  | * | *** |
|  | **CBTA-3** | *** | *** | *** | *** | *** | *** | *** | *** | ** | *** | *** |  |  | **CBT-3** | *** | *** | *** | *** | *** | *** | *** | *** |  |  |  |  |
| **(C)** | **373-12b** |  |  |  | ** |  |  | *** | *** | * |  | * | *** | **(F)** | **329-11b** | *** | *** | *** | *** | *** | *** | *** | *** |  |  |  | *** |
|  | **373-12c** | *** | *** | *** |  |  |  |  |  | *** | *** | *** |  |  | **329-11c** |  |  |  | *** |  |  |  |  |  |  |  | *** |
|  | **371-14a** |  |  |  |  |  |  |  |  |  |  |  |  |  | **327-13a** |  |  |  |  |  |  |  |  |  |  |  |  |
|  | **417-15a** | *** | *** | *** | *** | *** | *** | *** | *** | *** | *** | *** | *** |  | **373-15b** | *** | *** | * | *** | *** | *** | *** |  | * |  | * | *** |
|  | **357-16a** | * | * | * |  | * | ** | *** | *** |  |  |  | *** |  | **313-16b** | * |  |  | *** | *** | *** | * | *** | ** | ** | *** | *** |
|  | **375-19a** |  |  | *** | *** | *** | *** | *** |  | *** | *** | *** | *** |  | **331-18a** | *** | *** | * | *** | *** | *** | * | *** |  |  |  | *** |

^a^Two-way ANOVA followed by a Sidak post-hoc multiple comparisons test. A value of p ≤ 0.05 was considered significant for all tests.

**Table S15. Statistical significant differences in phytocannabinoid concentrations in Type III extracts dissolved in DMSO, ethanol and olive oil for different storage times (t_0_ versus t_12_^a^)**

|  |  | DMSO | | | | Ethanol | | | | Olive Oil | | | |  |  | DMSO | | | | Ethanol | | | | Olive Oil | | | |
| --- | --- | --- | --- | --- | --- | --- | --- | --- | --- | --- | --- | --- | --- | --- | --- | --- | --- | --- | --- | --- | --- | --- | --- | --- | --- | --- | --- |
|  |  | **Storage Temperature [°C]** | | | | **Storage Temperature [°C]** | | | | **Storage Temperature [°C]** | | | |  |  | **Storage Temperature [°C]** | | | | **Storage Temperature [°C]** | | | | **Storage Temperature [°C]** | | | |
|  |  | **-80** | **-30** | **4** | **25** | **-80** | **-30** | **4** | **25** | **-80** | **-30** | **4** | **25** |  |  | **-80** | **-30** | **4** | **25** | **-80** | **-30** | **4** | **25** | **-80** | **-30** | **4** | **25** |
| **(A)** | **CBGA** | *** | *** | *** | *** | ** | ** | ** | *** | ** | * | * | *** | **(D)** | **CBG** |  |  |  | *** | *** | *** | ** | *** | * | ** | * | *** |
|  | **CBGVA** |  |  |  |  |  |  |  |  |  |  |  |  |  | **CBGV** | * | * | * | *** |  |  | * | *** |  |  |  |  |
|  | **SesquiCBGA** |  |  |  |  |  |  |  |  |  |  |  |  |  | **SesquiCBG** | ** | *** | ** | ** | * | * | * | ** | ** | ** | ** |  |
|  | **Δ^9^-THCA** |  |  |  | *** |  |  |  | *** | * | * | * | *** |  | **Δ^9^-THC** |  |  | * |  |  | * |  | *** | * | * | ** |  |
|  | **Δ^9^-THCA-C4** |  |  |  |  |  |  |  |  |  |  |  |  |  | **Δ^9^-THC-C4** |  |  |  |  |  |  |  |  |  |  |  |  |
|  | **Δ^9^-THCVA** | * |  | ** | *** | * | * | * | *** | * | * | * | *** |  | **Δ^9^-THCV** | * | * |  | ** |  |  |  | * | * | * |  |  |
|  | **CBDA** | * | ** | ** | *** | * | * | * | *** |  |  |  | * |  | **CBD** | * | * | ** | *** |  |  |  | *** |  |  |  |  |
|  | **CBDA-C4** | * | * | ** | *** |  | * | * | *** |  |  |  |  |  | **CBD-C4** |  |  |  | *** | * | * | * | ** | * | * | * | * |
|  | **CBDVA** | *** | *** | *** | *** | ** | *** | *** | *** | * | * | * | ** |  | **CBDV** | *** | *** | *** | *** |  |  |  | *** | * | * | * |  |
|  | **CBDOA** | * |  | * | *** |  |  |  | *** |  |  |  |  |  | **CBDO** |  |  |  | *** |  |  |  | *** |  |  |  |  |
|  | **CBDMA** |  |  |  |  |  |  |  |  |  |  |  |  |  | **CBDM** |  |  |  |  |  |  |  | *** |  |  |  |  |
|  | **CBCA** |  |  |  | * | * | * | * | *** | * | * | * | * |  | **CBC** |  |  |  |  |  |  |  | *** |  |  |  |  |
|  | **CBCA-C4** |  |  |  |  |  |  |  |  |  |  |  |  |  | **CBC-C4** | ** | ** | *** | ** | *** | *** | *** | *** |  |  |  |  |
|  | **CBCVA** | *** | *** | *** | *** | *** | *** | *** | *** | *** | *** | *** | *** |  | **CBCV** |  |  |  |  |  |  |  | *** |  |  |  |  |
|  | **CBCOA** |  |  |  |  |  |  |  |  |  |  |  |  |  | **CBCO** |  |  |  |  |  |  |  | *** |  |  |  |  |
| **(B)** | **CBNA** |  |  | * | ** | *** | *** | *** | *** | *** | *** | *** | *** | **(E)** | **CBN** | *** | *** | *** | *** | *** | *** | *** | *** | *** | *** | *** | *** |
|  | **CBEA** |  |  | * | *** | * | ** | * | *** |  |  |  | *** |  | **CBE** |  |  |  |  |  |  |  | * |  |  |  |  |
|  | **CBEVA** |  |  |  |  |  |  |  |  |  | * |  | *** |  | **CBEV** |  |  |  |  |  |  |  |  |  |  |  |  |
|  | **CBNDA** |  |  |  |  |  |  |  |  |  |  |  |  |  | **CBND** |  | * | * | *** |  |  |  | *** |  |  |  |  |
|  | **CBTA-1** |  |  |  |  |  |  |  |  |  |  |  |  |  | **CBT-1** | * | ** | *** | *** |  |  |  |  |  |  |  | *** |
|  |  |  |  |  |  |  |  |  |  |  |  |  |  |  | **CBT-2** |  |  |  |  | * | * | * | *** |  |  |  | *** |
|  | **CBTA-3** |  | * | * |  |  |  |  |  |  |  |  |  |  | **CBT-3** | * |  |  | ** |  |  |  |  |  |  |  |  |
| **(C)** | **373-12a** | ** | * | *** | *** |  |  |  | *** |  |  |  | *** | **(F)** | **329-11a** | *** | *** | ** | *** | *** | *** | *** | * | *** | *** | *** | *** |
|  | **373-12b** | * | * | * | * | ** | ** | * | ** | * | * | * | * |  | **329-11b** |  |  |  | * |  | * |  |  |  |  |  | * |
|  | **373-12c** |  |  |  |  |  |  |  |  |  |  |  |  |  | **329-11c** |  |  |  |  |  |  |  |  |  |  |  | ** |
|  | **371-14a** |  |  |  |  |  |  | *** | * |  |  |  |  |  | **327-13a** |  |  |  |  |  |  | ** | *** |  |  |  |  |
|  | **417-15a** |  |  |  |  |  |  | * |  |  |  |  | *** |  | **373-15b** |  |  |  |  |  |  |  |  |  |  |  |  |
|  | **357-16a** | *** | *** | *** | *** | *** | *** | *** | * | *** | *** | *** | *** |  | **313-16b** | * | ** |  |  | * |  | * | *** | ** | ** | ** |  |
|  | **375-19a** |  |  | * | *** |  |  |  | *** |  |  |  | * |  | **331-18a** |  |  |  | *** | *** | *** | *** | * | ** | ** | ** | ** |

^a^One-way ANOVA followed by a Tukey post-hoc multiple comparisons test. A value of p ≤ 0.05 was considered significant for all tests.

**Table S16i. Statistical significant differences in phytocannabinoid concentrations in Type III extracts dissolved in DMSO and stored for one year for different storage temperatures (T_1_/T_2_)**

|  |  | **DMSO** | | | | | | | | | | | | | |
| --- | --- | --- | --- | --- | --- | --- | --- | --- | --- | --- | --- | --- | --- | --- | --- |
|  |  | **Comparison of storage temperatures^a^ [°C]** | | | | | |  |  | **Comparison of storage temperatures^a^ [°C]** | | | | | |
|  |  | **25/4** | **25/-30** | **25/-80** | **4/-30** | **4/-80** | **-30/-80** |  |  | **25/4** | **25/-30** | **25/-80** | **4/-30** | **4/-80** | **-30/-80** |
| **(A)** | **CBGA** | *** | *** | *** | * | * |  | **(D)** | **CBG** | *** | *** | *** |  |  |  |
|  | **CBGVA** | *** | *** | *** | * | * |  |  | **CBGV** | *** | *** | *** |  |  |  |
|  | **SesquiCBGA** | *** | *** | *** | * | * |  |  | **SesquiCBG** |  |  |  |  |  |  |
|  | **Δ^9^-THCA** | *** | *** | *** |  |  |  |  | **Δ^9^-THC** | ** |  | * | * |  |  |
|  | **Δ^9^-THCA-C4** |  | * | * |  |  |  |  | **Δ^9^-THC-C4** |  |  |  |  |  |  |
|  | **Δ^9^-THCVA** | *** | *** | *** | ** | * |  |  | **Δ^9^-THCV** | * |  |  |  |  |  |
|  | **CBDA** | *** | *** | *** |  |  |  |  | **CBD** | *** | *** | *** |  |  |  |
|  | **CBDA-C4** | *** | *** | *** | ** | * |  |  | **CBD-C4** | *** | *** | *** |  |  |  |
|  | **CBDVA** | *** | *** | *** | ** | * |  |  | **CBDV** | *** | *** | *** |  |  |  |
|  | **CBDOA** | *** | *** | *** | *** | ** |  |  | **CBDO** | *** | *** | *** |  |  |  |
|  | **CBDMA** | *** | *** | *** | * | * |  |  | **CBDM** |  |  |  |  |  |  |
|  | **CBCA** | ** | *** | *** |  |  |  |  | **CBC** | *** | ** | *** | * | * |  |
|  | **CBCA-C4** | * | ** | * |  |  |  |  | **CBC-C4** |  |  |  |  |  |  |
|  | **CBCVA** | *** | *** | *** |  |  |  |  | **CBCV** | *** | *** | *** |  |  |  |
|  | **CBCOA** |  |  | * |  | * |  |  | **CBCO** |  |  |  |  |  |  |
| **(B)** | **CBNA** | * | ** | ** | * |  |  | **(E)** | **CBN** | * |  |  | * |  |  |
|  | **CBEA** | *** | *** | *** | * | * |  |  | **CBE** | * | * |  | *** | ** |  |
|  | **CBEVA** |  | * | * | * |  |  |  | **CBEV** |  |  |  |  |  |  |
|  | **CBNDA** | *** | *** | *** | * | * |  |  | **CBND** | *** | *** | *** |  |  |  |
|  | **CBTA-1** |  |  |  |  |  |  |  | **CBT-1** | *** | *** | *** | * | * |  |
|  |  |  |  |  |  |  |  |  | **CBT-2** |  |  |  |  |  |  |
|  | **CBTA-3** |  | * |  |  |  |  |  | **CBT-3** | *** | *** | *** |  |  |  |
| **(C)** | **373-12a** | *** | *** | *** | *** | *** |  | **(F)** | **329-11a** | *** | *** | *** |  |  |  |
|  | **373-12b** |  | * |  |  |  |  |  | **329-11b** | *** | *** | *** |  |  |  |
|  | **373-12c** | *** | ** | ** | *** | *** |  |  | **329-11c** |  | * |  | * |  |  |
|  | **371-14a** |  | ** | * | * | * |  |  | **327-13a** |  |  |  |  |  |  |
|  | **417-15a** |  |  |  |  |  |  |  | **373-15b** | ** | *** | *** | *** | *** |  |
|  | **357-16a** | *** | *** | *** | ** | ** |  |  | **313-16b** |  | ** | ** | ** | ** |  |
|  | **375-19a** | *** | *** | *** | *** | *** |  |  | **331-18a** | *** | *** | *** |  |  |  |

^a^One-way ANOVA followed by a Tukey post-hoc multiple comparisons test. A value of p ≤ 0.05 was considered significant for all tests.

**Table S16ii. Statistical significant differences in phytocannabinoid concentrations in Type III extracts dissolved in ethanol and stored for one year for different storage temperatures (T_1_/T_2_)**

|  |  | **Ethanol** | | | | | | | | | | | | | |
| --- | --- | --- | --- | --- | --- | --- | --- | --- | --- | --- | --- | --- | --- | --- | --- |
|  |  | **Comparison of storage temperatures^a^ [°C]** | | | | | |  |  | **Comparison of storage temperatures^a^ [°C]** | | | | | |
|  |  | **25/4** | **25/-30** | **25/-80** | **4/-30** | **4/-80** | **-30/-80** |  |  | **25/4** | **25/-30** | **25/-80** | **4/-30** | **4/-80** | **-30/-80** |
| **(A)** | **CBGA** | *** | *** | *** |  |  |  | **(D)** | **CBG** | *** | *** | *** |  |  |  |
|  | **CBGVA** | *** | *** | *** |  |  |  |  | **CBGV** | *** | *** | *** |  |  |  |
|  | **SesquiCBGA** | *** | *** | *** |  |  |  |  | **SesquiCBG** |  |  |  |  |  |  |
|  | **Δ^9^-THCA** | *** | *** | *** |  |  |  |  | **Δ^9^-THC** | *** | *** | *** |  |  |  |
|  | **Δ^9^-THCA-C4** | *** | *** | *** |  | * | * |  | **Δ^9^-THC-C4** |  |  |  |  |  |  |
|  | **Δ^9^-THCVA** | *** | *** | *** |  |  |  |  | **Δ^9^-THCV** |  |  |  |  |  |  |
|  | **CBDA** | *** | *** | *** |  |  |  |  | **CBD** | *** | *** | *** | * | * |  |
|  | **CBDA-C4** | *** | *** | *** |  |  |  |  | **CBD-C4** | *** | *** | *** |  |  |  |
|  | **CBDVA** | *** | *** | *** |  |  | * |  | **CBDV** | *** | *** | *** | * |  |  |
|  | **CBDOA** | *** | *** | *** |  |  |  |  | **CBDO** | *** | *** | *** |  |  |  |
|  | **CBDMA** | *** | *** | *** |  |  |  |  | **CBDM** | *** | *** | *** |  |  |  |
|  | **CBCA** | *** | *** | *** |  |  |  |  | **CBC** | *** | *** | *** |  |  |  |
|  | **CBCA-C4** | *** | *** | *** |  |  |  |  | **CBC-C4** | *** | *** | *** |  |  |  |
|  | **CBCVA** |  |  |  |  |  |  |  | **CBCV** | *** | *** | *** |  |  |  |
|  | **CBCOA** |  |  |  |  |  |  |  | **CBCO** | *** | *** | *** |  |  |  |
| **(B)** | **CBNA** | *** | *** | *** |  |  | * | **(E)** | **CBN** | *** | *** | *** |  |  |  |
|  | **CBEA** | *** | *** | *** |  |  |  |  | **CBE** | *** | *** | *** |  |  |  |
|  | **CBEVA** | *** | ** | *** |  |  |  |  | **CBEV** |  |  |  |  |  |  |
|  | **CBNDA** | *** | *** | *** |  |  |  |  | **CBND** | *** | *** | *** |  |  |  |
|  | **CBTA-1** |  |  |  |  |  |  |  | **CBT-1** | *** | *** | *** |  |  |  |
|  |  |  |  |  |  |  |  |  | **CBT-2** | *** | *** | *** |  |  |  |
|  | **CBTA-3** | *** | *** | *** |  |  |  |  | **CBT-3** |  |  |  |  |  |  |
| **(C)** | **373-12a** | *** | *** | *** |  |  | * | **(F)** | **329-11a** | *** | *** | *** |  |  |  |
|  | **373-12b** | *** | *** | *** |  |  |  |  | **329-11b** | *** | *** | *** |  |  |  |
|  | **373-12c** | *** | *** | *** |  |  |  |  | **329-11c** | *** | *** | *** |  |  |  |
|  | **371-14a** | *** | *** | *** | *** | *** |  |  | **327-13a** | *** | *** | *** | * | * |  |
|  | **417-15a** | *** | * | * | *** | *** |  |  | **373-15b** | *** | *** | *** | *** | *** |  |
|  | **357-16a** | *** | *** | *** |  |  |  |  | **313-16b** | ** | *** | ** |  |  |  |
|  | **375-19a** | *** | *** | *** |  |  |  |  | **331-18a** | *** | *** | *** |  |  |  |

^a^One-way ANOVA followed by a Tukey post-hoc multiple comparisons test. A value of p ≤ 0.05 was considered significant for all tests.

**Table S16iii. Statistical significant differences in phytocannabinoid concentrations in Type III extracts dissolved in olive oil and stored for one year for different storage temperatures (T_1_/T_2_)**

|  |  | **Olive Oil** | | | | | | | | | | | | | |
| --- | --- | --- | --- | --- | --- | --- | --- | --- | --- | --- | --- | --- | --- | --- | --- |
|  |  | **Comparison of storage temperatures^a^ [°C]** | | | | | |  |  | **Comparison of storage temperatures [°C]** | | | | | |
|  |  | **25/4** | **25/-30** | **25/-80** | **4/-30** | **4/-80** | **-30/-80** |  |  | **25/4** | **25/-30** | **25/-80** | **4/-30** | **4/-80** | **-30/-80** |
| **(A)** | **CBGA** | *** | *** | *** |  |  |  | **(D)** | **CBG** | *** | *** | *** |  |  |  |
|  | **CBGVA** | * | * | * |  |  |  |  | **CBGV** |  |  |  |  |  |  |
|  | **SesquiCBGA** | ** | * |  |  | * |  |  | **SesquiCBG** | *** | *** | *** |  |  |  |
|  | **Δ^9^-THCA** | * | ** | * |  |  |  |  | **Δ^9^-THC** | * | * | * |  |  |  |
|  | **Δ^9^-THCA-C4** |  |  |  |  |  |  |  | **Δ^9^-THC-C4** |  |  |  |  |  |  |
|  | **Δ^9^-THCVA** | *** | *** | *** |  |  |  |  | **Δ^9^-THCV** |  | * | * |  |  |  |
|  | **CBDA** | *** | *** | *** |  |  |  |  | **CBD** | *** | *** | *** |  |  |  |
|  | **CBDA-C4** | * | ** | * |  |  |  |  | **CBD-C4** | ** | ** | ** |  |  |  |
|  | **CBDVA** | *** | *** | *** |  |  |  |  | **CBDV** | *** | * | * | * | * |  |
|  | **CBDOA** | * | * | * |  |  |  |  | **CBDO** |  |  |  |  |  |  |
|  | **CBDMA** | * | ** | * |  |  |  |  | **CBDM** |  |  |  |  |  |  |
|  | **CBCA** | ** | ** | * | * |  |  |  | **CBC** |  |  |  |  |  |  |
|  | **CBCA-C4** | * | ** | * |  |  |  |  | **CBC-C4** |  |  |  |  |  |  |
|  | **CBCVA** |  |  |  |  |  |  |  | **CBCV** |  |  |  |  |  |  |
|  | **CBCOA** |  |  |  |  |  |  |  | **CBCO** |  |  |  |  |  |  |
| **(B)** | **CBNA** | *** | * | ** |  |  |  | **(E)** | **CBN** | *** | *** | *** |  |  |  |
|  | **CBEA** | *** | *** | *** |  |  | * |  | **CBE** |  |  |  |  |  |  |
|  | **CBEVA** | *** | *** | *** | * |  | * |  | **CBEV** |  |  |  |  |  |  |
|  | **CBNDA** | *** | *** | *** |  | * | * |  | **CBND** |  |  |  |  |  |  |
|  | **CBTA-1** |  |  |  |  |  |  |  | **CBT-1** | *** | *** | *** |  |  |  |
|  |  |  |  |  |  |  |  |  | **CBT-2** | ** | ** | *** |  |  |  |
|  | **CBTA-3** | * | * | * |  |  |  |  | **CBT-3** | *** | *** | *** |  |  |  |
| **(C)** | **373-12a** | *** | *** | *** |  |  |  | **(F)** | **329-11a** | *** | *** | *** |  |  |  |
|  | **373-12b** |  |  | * |  |  |  |  | **329-11b** | ** | ** | ** |  |  |  |
|  | **373-12c** |  |  |  |  |  |  |  | **329-11c** | *** | *** | *** |  |  |  |
|  | **371-14a** |  |  |  |  |  |  |  | **327-13a** |  |  |  |  |  |  |
|  | **417-15a** | *** | *** | *** |  |  |  |  | **373-15b** | *** | *** | *** | *** | *** | *** |
|  | **357-16a** | *** | *** | *** |  |  | * |  | **313-16b** | *** | ** | ** |  |  |  |
|  | **375-19a** | *** | *** | *** |  |  |  |  | **331-18a** | * | * | * |  |  |  |

^a^One-way ANOVA followed by a Tukey post-hoc multiple comparisons test. A value of p ≤ 0.05 was considered significant for all tests.

**Table S17. Statistical significant differences in phytocannabinoid concentrations in Type III extracts following one year of storage for different storage solvents**

|  |  | DMSO vs. Ethanol**^a^** | | | | DMSO vs. Olive Oil**^a^** | | | | Ethanol vs. Olive Oil**^a^** | | | |  |  | DMSO vs. Ethanol**^a^** | | | | DMSO vs. Olive Oil**^a^** | | | | Ethanol vs. Olive Oil**^a^** | | | |
| --- | --- | --- | --- | --- | --- | --- | --- | --- | --- | --- | --- | --- | --- | --- | --- | --- | --- | --- | --- | --- | --- | --- | --- | --- | --- | --- | --- |
|  |  | **Storage Temperature [°C]** | | | | **Storage Temperature [°C]** | | | | **Storage Temperature [°C]** | | | |  |  | **Storage Temperature [°C]** | | | | **Storage Temperature [°C]** | | | | **Storage Temperature [°C]** | | | |
|  |  | **-80** | **-30** | **4** | **25** | **-80** | **-30** | **4** | **25** | **-80** | **-30** | **4** | **25** |  |  | **-80** | **-30** | **4** | **25** | **-80** | **-30** | **4** | **25** | **-80** | **-30** | **4** | **25** |
| **(A)** | **CBGA** | ** |  | *** | * | *** | *** | *** | *** | * | *** | ** | *** | **(D)** | **CBG** | *** | *** | *** | *** | *** | *** | *** | *** | * | * | * | *** |
|  | **CBGVA** |  | * | * | ** |  |  | *** | *** |  | ** | * | *** |  | **CBGV** |  | *** | * | * | *** | *** | *** | *** | *** | *** | *** | *** |
|  | **SesquiCBGA** | *** |  | *** | *** | *** | *** | *** | *** |  | *** | *** | *** |  | **SesquiCBG** | *** | *** | *** |  | * | *** | * | *** |  |  |  | *** |
|  | **Δ^9^-THCA** |  |  |  | *** | *** | *** | *** | * | *** | *** | *** | *** |  | **Δ^9^-THC** |  |  | ** | *** | *** | * | *** |  | *** | *** | *** | *** |
|  | **Δ^9^-THCA-C4** |  |  | * |  | *** | *** | * |  | *** | *** | *** |  |  | **Δ^9^-THC-C4** |  |  |  |  |  |  |  |  |  |  |  |  |
|  | **Δ^9^-THCVA** |  |  | *** | * |  | ** | * | *** | ** | * |  | *** |  | **Δ^9^-THCV** |  |  |  |  |  |  |  |  |  |  |  | * |
|  | **CBDA** | ** | *** | *** | * | *** | *** | *** | *** | *** | *** | *** | *** |  | **CBD** | *** | *** | *** | * | *** | *** | *** | *** |  |  |  | *** |
|  | **CBDA-C4** | * |  | *** | ** | *** | *** | *** | *** | *** | *** | *** | *** |  | **CBD-C4** | *** | *** | *** | *** | *** | *** | *** | *** |  |  |  | *** |
|  | **CBDVA** | * |  | *** | ** | *** | *** | *** | *** | *** | *** | *** | *** |  | **CBDV** | *** | *** | *** | *** | *** | *** | *** | *** | *** | *** | *** | *** |
|  | **CBDOA** | * |  | *** |  | *** | *** | *** | *** | *** | *** | *** | *** |  | **CBDO** |  |  |  | *** |  |  |  | *** |  |  |  | *** |
|  | **CBDMA** | ** |  | *** |  | *** | *** | *** | *** | * | *** | * | *** |  | **CBDM** |  |  |  | *** |  |  |  |  |  |  |  | *** |
|  | **CBCA** | *** | *** | *** | *** | *** | *** | ** |  |  | *** | *** | *** |  | **CBC** | *** |  | *** | *** | *** | *** | *** |  | * | *** | * | *** |
|  | **CBCA-C4** | *** | *** | *** | *** | *** | *** | *** |  |  | * |  | *** |  | **CBC-C4** | *** | *** | *** | * | *** | *** | *** | *** | ** | * | ** | *** |
|  | **CBCVA** | *** | *** | *** | *** | *** | *** | *** | *** |  |  |  |  |  | **CBCV** |  |  |  | *** |  |  |  |  |  |  |  | *** |
|  | **CBCOA** | *** |  |  |  | *** |  |  |  |  |  |  |  |  | **CBCO** |  |  |  | *** |  |  |  |  |  |  |  | *** |
| **(B)** | **CBNA** | *** | *** | *** | *** | *** | *** | *** |  | * |  | * | *** | **(E)** | **CBN** |  | *** |  | *** |  | * | * | *** |  |  |  |  |
|  | **CBEA** | *** | *** |  |  |  | ** | ** | *** | ** | *** | *** | *** |  | **CBE** | *** | *** | * | *** | *** | *** | *** | *** | *** | * | ** | *** |
|  | **CBEVA** | *** | *** | * | ** |  |  |  | *** | ** | *** | *** | *** |  | **CBEV** |  |  |  |  |  |  |  |  |  |  |  |  |
|  | **CBNDA** | *** | *** | *** |  | *** | *** | *** | *** | *** | *** | *** | *** |  | **CBND** | * | *** | *** | *** | * | *** | *** | *** |  |  |  | *** |
|  | **CBTA-1** |  |  |  |  |  |  |  |  |  |  |  |  |  | **CBT-1** | *** | *** | *** | *** | *** | *** | *** | *** |  | * | * | *** |
|  |  |  |  |  |  |  |  |  |  |  |  |  |  |  | **CBT-2** |  |  |  |  |  |  |  | * |  |  |  |  |
|  | **CBTA-3** | *** | *** | *** | *** | ** | *** | ** |  | ** | *** | *** | *** |  | **CBT-3** | *** | *** | *** | *** | *** | *** | *** | *** | * | * | * | *** |
| **(C)** | **373-12a** | *** | *** | *** | * | *** | *** | *** | *** |  | ** | * | *** | **(F)** | **329-11a** | *** | *** | *** | *** | *** | *** | *** | *** | * | ** | * | *** |
|  | **373-12b** | *** | *** | *** | *** | *** | * | * |  | *** | *** | *** | *** |  | **329-11b** | * | ** | * | ** | * |  |  |  |  |  |  | *** |
|  | **373-12c** |  |  |  |  |  |  |  |  |  |  |  |  |  | **329-11c** |  | *** |  | *** |  |  | *** | *** | *** | *** | *** | *** |
|  | **371-14a** | *** | ** | *** | *** |  |  | *** | *** | *** | *** | *** | *** |  | **327-13a** | *** | *** | *** | *** |  |  |  |  | *** | *** | *** | *** |
|  | **417-15a** |  |  | *** | * |  |  |  | *** |  |  | * | *** |  | **373-15b** | *** | *** | *** | *** | *** | *** | *** | *** | *** | *** | *** | *** |
|  | **357-16a** | *** | *** | *** |  | *** | *** | *** | *** |  | ** | * | *** |  | **313-16b** |  | ** | * | *** |  |  | *** |  | * | *** | *** | *** |
|  | **375-19a** | *** | ** | *** | *** | *** | *** | *** | *** | * | *** | ** | *** |  | **331-18a** | *** | *** | *** | *** | *** | *** | *** | *** | *** | *** | ** | *** |

^a^Two-way ANOVA followed by a Sidak post-hoc multiple comparisons test. A value of p ≤ 0.05 was considered significant for all tests.
